# Emerging Roles of Macrophage Polarization in Osteoarthritis: Mechanisms and Therapeutic Strategies

**DOI:** 10.1111/os.13993

**Published:** 2024-01-31

**Authors:** Zimu Yuan, Decheng Jiang, Mengzhu Yang, Jie Tao, Xin Hu, Xiao Yang, Yi Zeng

**Affiliations:** ^1^ West China Medical School Sichuan University Chengdu China; ^2^ West China Hospital Sichuan University Chengdu China; ^3^ Orthopedic Research Institute, Department of Orthopedics West China Hospital, Sichuan University Chengdu China; ^4^ National Engineering Research Center for Biomaterials Sichuan University Chengdu China

**Keywords:** Macrophage Polarization, Osteoarthritis, Signaling Pathway

## Abstract

Osteoarthritis (OA) is the most common chronic degenerative joint disease in middle‐aged and elderly people, characterized by joint pain and dysfunction. Macrophages are key players in OA pathology, and their activation state has been studied extensively. Various studies have suggested that macrophages might respond to stimuli in their microenvironment by changing their phenotypes to pro‐inflammatory or anti‐inflammatory phenotypes, which is called macrophage polarization. Macrophages accumulate and become polarized (M1 or M2) in many tissues, such as synovium, adipose tissue, bone marrow, and bone mesenchymal tissues in joints, while resident macrophages as well as other stromal cells, including fibroblasts, chondrocytes, and osteoblasts, form the joint and function as an integrated unit. In this study, we focus exclusively on synovial macrophages, adipose tissue macrophages, and osteoclasts, to investigate their roles in the development of OA. We review recent key findings related to macrophage polarization and OA, including pathogenesis, molecular pathways, and therapeutics. We summarize several signaling pathways in macrophage reprogramming related to OA, including NF‐κB, MAPK, TGF‐β, JAK/STAT, PI3K/Akt/mTOR, and NLRP3. Of note, despite the increasing availability of treatments for osteoarthritis, like intra‐articular injections, surgery, and cellular therapy, the demand for more effective clinical therapies has remained steady. Therefore, we also describe the current prospective therapeutic methods that deem macrophage polarization to be a therapeutic target, including physical stimulus, chemical compounds, and biological molecules, to enhance cartilage repair and alleviate the progression of OA.

## Graphical abstract

Macrophages are key players in osteoarthritis (OA) pathology. In this review, we focus on synovial macrophages, adipose tissue macrophages, and osteoclasts, to investigate their roles in the development of OA. We review recent key findings related to macrophage polarization and OA, including pathogenesis, molecular pathways, and novel therapeutics.

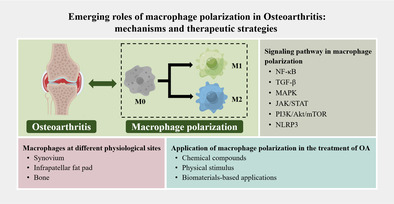

## Introduction

Osteoarthritis (OA) is the most common chronic degenerative joint disease in middle‐aged and elderly people, characterized by joint pain and dysfunction.^1^, ^2^ It is estimated that 240 million individuals worldwide have symptomatic OA currently, with women aged 60 and older having a 1.80‐fold higher prevalence than men.^3^ Several risk factors have been reported to be associated with OA development, including gender, age, hereditary factors, diabetes, and obesity.^4^, ^5^, ^6^, ^7^, ^8^ In addition, the prevalence of OA is related to one's lifestyle and habits.^9^ Unfortunately, the prevalence of OA has been increasing in younger people in recent years, with a large increase in the proportion of patients under 65 years old.^10^ Thereafter, more and more people suffer from OA, which has a negative effect on the quality of their later life, resulting in a huge burden for individuals, families, and society.

It is widely acknowledged that inflammation plays a vital role in the development of OA. Macrophages, a kind of innate immune cells, regulate the immune and inflammatory process in the physiological state of a variety of diseases. Macrophages can be classified into two subtypes: activated macrophages (M1) and the other alternatively activated macrophages (M2). Generally, M1 is a kind of proinflammatory cell, whereas M2 plays opposite roles in the inflammation process. The transformation of macrophages from M1 to M2 in response to inflammatory stimuli is known as macrophage polarization, involved in the pathophysiological processes of many disorders, such as autoimmune diseases,^11^ infections,^12^ liver diseases,^13^ tumors,^14^ and OA.

Herein, we review recent key findings related to macrophage polarization and OA, including pathogenesis, molecular pathways, and therapeutics. After scanning, we summarize the macrophage polarization and underlying potential mechanism in different joint tissues such as synovium, infrapatellar fat pad (IPFP), and bone during the progression of OA. Despite the increasing availability of treatments for OA, like intra‐articular (IA) injections, surgery, and cellular therapy, the demand for more effective clinical therapies remains high. Therefore, we also describe the current prospective therapeutic methods that deem macrophage polarization as a therapeutic target to enhance cartilage repair and alleviate the progression of OA.

## Macrophage Classification in Osteoarthritis

Osteoarthritis is a disease that affects most joint tissues. Inflammation and the most abundant immune cell type within the joint, macrophages, have been recognized as possible players in disease development and progression. In the context of macrophage origin, macrophages emerge from two distinct lineages. Tissue‐resident macrophages exhibit local self‐renewal, independent of adult hematopoiesis,^15^, ^16^, ^17^ while short‐lived monocyte‐derived macrophages originate from adult hematopoietic stem cells, predominantly accumulating in inflamed lesions.^18^ The precise contribution of these macrophage lineages to the progression of OA remains unclear.

Numerous investigations have proposed that macrophages undergo phenotypic alterations toward proinflammatory or anti‐inflammatory states in response to diverse forms of functional activation dictated by signals within their microenvironment.^19^, ^20^, ^21^ Specific phenotypes of macrophages might differentially modulate the anabolic or catabolic responses of different cell types with the onset or progression of OA.

### Polarized Macrophage Classification

Broadly, macrophages are commonly categorized into two functionally activated forms, often denoted as exhibiting an M1 or M2 phenotype.^22^ These distinct phenotypes play divergent roles in either the initiation or advancement of OA.

#### M1 Macrophages

M1 macrophages, or classically activated macrophages, serve as antimicrobial and pro‐inflammatory players, which are activated in response to stimuli from T helper type 1 (Th1) cells.^23^ M1 macrophages are usually activated by pattern recognition receptors (PRR), such as Toll‐like receptors (TLRs) and NOD‐like receptors (NLRs), when they recognize pathogen‐associated molecular patterns (PAMPs) and damage‐associated molecular patterns (DAMPs). Thereafter, this phenotype initiates the downstream inflammatory signaling pathways, such as nuclear factor (NF)‐κB signaling, thereby inducing a massive release of pro‐inflammatory cytokines and chemokines.^24^, ^25^

Studies have shown that enhanced synovial M1 macrophage polarization is responsible for the increased severity of OA.^26^ The destruction of cartilage mediated by M1 macrophages in the progression of OA has been highlighted in previous studies. Fahy *et al*.^27^ stated that the M1‐associated cytokines IL‐6, IL‐1b, TNF‐α, and oncostatin M (OSM) induced destructive processes in chondrocytes, including downregulation of collagen type II and aggrecan synthesis. In addition, synovial M1 macrophages were also shown to upregulate the production of proteolytic enzymes, such as matrix metalloproteinase (MMP)‐1, MMP3, MMP13, MMP9 aggrecanases, and cyclooxygenase‐2, which contribute to articular degeneration.^28^, ^29^ Inflammation of synovial membranes might be another etiology in which M1 macrophages participate. Sun *et al*. showed that obesity, as a generally acknowledged pathogenic factor for OA, is associated with spontaneous and local inflammation of the synovial membranes, which was followed by a predominant elevation of pro‐inflammatory M1 macrophages and increased synovitis.^30^, ^31^ All of these abovementioned studies seem to prove that the promotion of M1 macrophage polarization can aggravate OA progression.^26^ However, conditional macrophage deletion has been demonstrated to have no effect on the development of OA.^32^ Interventions for macrophage M1 polarization to attenuate the severity of OA are awaiting investigation.

It is worth mentioning that an M1 macrophage‐provoked excessive inflammatory response usually results in tissue destruction, which manifests as joint pain in OA patients. Li *et al*. found that IL‐1β, IL‐6, and TNF‐α played critical roles in pain in the early stages of knee OA and were correlated with pain.^33^ Because pain is one of the most prominent symptoms of OA and a primary motivator of clinical decision‐making, etiological research into pain is crucial not just for understanding OA but also for developing new medications to relieve it.

#### M2 Macrophages

M2 macrophages, also known as selectively activated macrophages, are primarily activated by “M2‐related” polarizing factors such as IL‐4, IL‐10, TGF‐β, and dexamethasone,^34^ which have pro‐healing or anti‐inflammatory effects. M2 macrophages are further subdivided into M2a, M2b, and M2c, which exhibit anti‐inflammatory characteristics and contribute to tissue repair and remodeling.^35^ Activation of mechanistic target of rapamycin complex 1 (mTORC1) increased synovial M1 macrophage polarization, which exacerbated cartilage degeneration and osteophyte formation in experimental OA models. Inhibition of mTORC1, in contrast, increased M2 polarization and attenuated OA development.^26^

In recent years, much progress has been made regarding the mechanism of macrophage polarization, which is a promising strategy in the treatment of OA. Zheng *et al*. reported that IA administration of D0 exosomes could lead to mitochondrial dysfunction and polarize macrophage response toward an M2 phenotype, thereby successfully preventing the development of OA.^36^ Bailey *et al*. reported that in macrophage‐depleted macrophage Fas‐induced apoptosis (MaFIA) mice, macrophage polarity shifted to the dominance of M1 macrophages and reduction of M2 macrophages in the synovial stroma, indicating a shift in the M1/M2 macrophage ratio in the joint following injury.^37^ However, macrophage depletion of both M1 and M2 subtypes has a confusing effect on OA progression. Wu *et al*. demonstrated that macrophage depletion by a small molecule (AP20187) in a MaFIA‐transgenic mouse model exhibited decreased osteophyte formation immediately following depletion but did not attenuate the severity of OA.^32^ Those studies indicated that the failure to transform from the M1 to M2 subtype might play a larger role in the progression of OA than the quantity of activated macrophages.

### Macrophages at Different Physiological Sites

**FIGURE 1:**
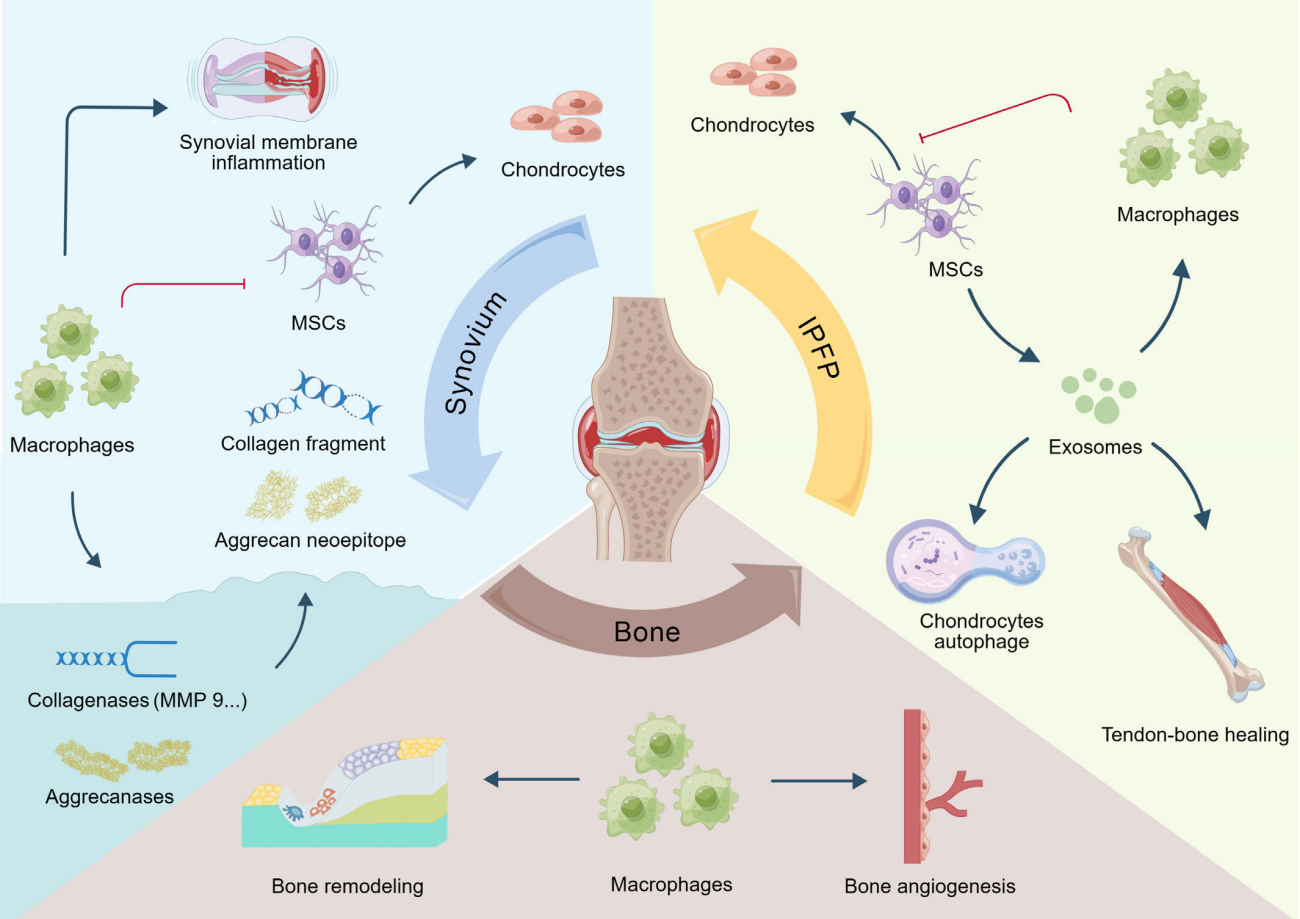
Polarized macrophages in different parts of the knee joint with osteoarthritis (OA).

Macrophages accumulate and become polarized (M1 or M2) in many tissues such as synovium, adipose tissue, bone marrow, mucosa, and bone mesenchymal tissues in joints, which comprise the appendicular skeleton, synovium, cartilage, tendons, ligaments, joint capsules, and their associated lymphatics and vasculature, while resident macrophages and other stromal cells, including fibroblasts, endothelial cells, chondrocytes, and osteoblasts, form the joint and function as an integrated unit.^38^ In the following sections, we focus exclusively on synovial macrophages, adipose tissue macrophages, and osteoclasts (Figure 1).

#### Synovium

The synovial membrane has a two‐layer structure, which is divided into the inner surface layer and the bottom layer. The inner surface layer is composed of two to three layers of macrophages and fibro‐like synovial cells, wherein the synovial cells can secrete synovial fluid and the macrophages can phagocytic worn debris. The bottom layer consists of fibrous connective tissue, blood vessels, a few lymphocytes, and macrophages.

The role of polarized macrophages in synovium tissue during the process of OA has been highlighted. It has been demonstrated that M1 macrophage polarization in the synovium exacerbates the progression of OA.^39^ In investigating the mechanism involved in this macrophage‐driven degradative progression, Bondeson and his colleagues discovered that depletion of macrophages in OA synovial explants significantly reduced the production of several cytokines, including TNF, IL‐1, IL‐6, and IL‐8. Importantly, macrophage depletion and neutralization of macrophage‐derived TNF and IL‐1 could downregulate MMP production, thereby linking synovial macrophages to cartilage degradation.^40^ Moreover, Blom *et al*. confirmed that synovial macrophages were involved in MMP‐mediated cartilage degeneration in the murine collagenase‐induced model of OA.^41^ These findings could indicate that OA synovial macrophages play an important role in perpetuating the production of proinflammatory cytokines and destructive enzymes. In recent years, further studies have been conducted to explore the roles and regulatory mechanisms of synovial macrophages and their polarization in the development of OA. Sun *et al*. (2022) reported that synovial M1 macrophage polarization was stimulated by mTORC1 activation and, in turn, exacerbated cartilage degeneration and osteophyte formation in experimental OA, whereas M2 polarization was enhanced by mTORC1 inhibition and attenuated OA development.^35^ Synovial macrophages, which serve as modulators and producers of nerve growth factor (NGF) in joint synovial tissue, have been shown to be associated with pain sensitivity in OA joints.^42^ Further, it is reported that the role of synovial macrophages is regulated by TNF‐α, which can alleviate joint pain in OA by upregulating the NGF signal transduction produced by synovial macrophages in OA joints.^43^

In addition to synovium tissue, macrophages were also detected in the synovial fluid of OA patients.^44^ Kraus *et al*. detected M1 (iNOS) and/or M2 (TGF‐b) macrophage markers in synovial fluid cells of two OA patients by immunostaining.^21^ Kulkarni *et al*. (2022) induced U937 cells with synovial fluid of progressive KL grades and indicated that synovial fluid in OA joints acted as a niche and facilitated M1 phenotype polarization by providing a proinflammatory microenvironment.^45^ Using transgenic mouse models with enhanced M1‐ or M2‐polarized macrophages, Zhang *et al*. showed that synovial macrophage M1 polarization exacerbated experimental collagenase‐induced OA while M2 polarization attenuated OA development.^26^ The findings of the above mentioned studies reveal the critical role of synovial M1 and M2 macrophages in the development of OA.

#### Infrapatellar Fat Pad

The infrapatellar fat pad (IPFP), also known as Hoffa's fat pad, is located below the patella and fills the potential space between the posterior patellar tendon, the condyle of the femur, and the anterior tibial plateau. It is a structure of extra‐synovial fat tissue within the bursae of the knee joint, composed of adipose tissue similar to subcutaneous fat. IPFP acts as an important organizational structure involved in the occurrence and development of OA,^46^ and the resident macrophages of IPFP contribute to the onset and progression of inflammatory joint diseases.^47^

The macrophages in IPFP participate in the pathogenesis of OA and other joint diseases. Not only the increasing number of macrophages but also M1‐like (CD11c^+^) and M2‐like (CD206^+^) macrophage markers have been reported in this tissue.^48^, ^49^ People with IPFP signal intensity alteration have a high risk of accelerated OA, which is characterized by local inflammation.^50^ Wei *et al*. suggested that macrophages harvested from the IPFP of diseased joints inhibit chondrogenesis of mesenchymal stem cells (MSCs),^51^ supporting the notion that IPFP macrophages play a potentially detrimental role in cartilage regeneration. Recent studies showed that the exosomes derived from several types of MSCs could maintain chondrocyte homeostasis and ameliorate the pathological severity of OA.^52^, ^53^ In an acute synovial/IPFP inflammation rat model, IPFP‐MSC exosome therapeutic treatment resulted in robust macrophage polarization toward an anti‐inflammatory therapeutic M2 phenotype within the synovium/IFP tissues.^47^ Wu *et al*. showed that miR‐100‐5p‐abundant exosomes derived from IPFP‐MSCs promote autophagy of chondrocytes through mTOR inhibition.^54^ IPFP MSC‐derived exosomes accelerate tendon–bone healing and IA graft remodeling after anterior cruciate ligament reconstruction, which might result from the immunomodulation of macrophage polarization.^55^

#### Bone

According to earlier OA research, cartilage breakdown is no longer the only pathogenic alteration in OA. In contrast, it is a whole joint condition encompassing cartilage as well as non‐cartilage tissues such as subchondral bone. Microstructural analysis suggested that cartilage and bone alterations occur concurrently,^56^ while analysis of the biomechanical properties of subchondral bone revealed that bone changes might occur before cartilage damage.^57^ Using novel microstructural analysis techniques, a comparative study by Chen *et al*. identified a drastic loss of rod‐like trabeculae and thickening of plate‐like trabeculae in all regions of the tibial plateau even if the cartilage was still intact.^58^ Thus, it is conceivable that changes in bone density and/or bone microstructure could be early pathologic characteristics of OA.

In the subchondral bone, rapid bone loss after traumatic injuries and bone sclerosis at the end stage are well‐recognized hallmarks of OA. There is a large body of evidence supporting that enhanced subchondral bone turnover plays an active and pivotal role in the onset and progression of OA in which the subchondral bone compartment undergoes active remodeling, a process that is partially influenced by macrophages.^59^, ^60^ A study in 2020 showed that abnormally increased platelet‐derived growth factor (PDGF)‐BB secretion by mononuclear preosteoclasts induced subchondral bone angiogenesis, which contributed to OA development.^61^ Another study demonstrated that platelet‐related scaffold for tissue engineering could promote osteochondral repair through immune regulation by M2 polarization and is a potential candidate for osteochondral tissue engineering.^62^ These findings indicated that some newly developed molecules or materials targeted at macrophage polarization might be effective biological reagents to prevent and treat OA in the clinic.

## Signaling Pathways in Macrophage Polarization

Signaling pathways are essential for OA development. A growing body of literature has clarified the effect of signaling pathways in OA and provides evidence that targeting these axes might be a viable therapeutic approach. However, it seems that simply activating or inhibiting these signaling pathways could be a double‐edged sword, with unavoidable side effects.

As mentioned before, macrophage polarization is dynamically affected by the various stimuli in the microenvironment. However, the underlying mechanism of this process remains obscure. Understanding the regulators and effectors of these signaling pathways on macrophage polarization would be useful to precisely target the polarized macrophage subsets, resulting in improved therapeutic efficacy with less toxicity in OA treatment.

**FIGURE 2:**
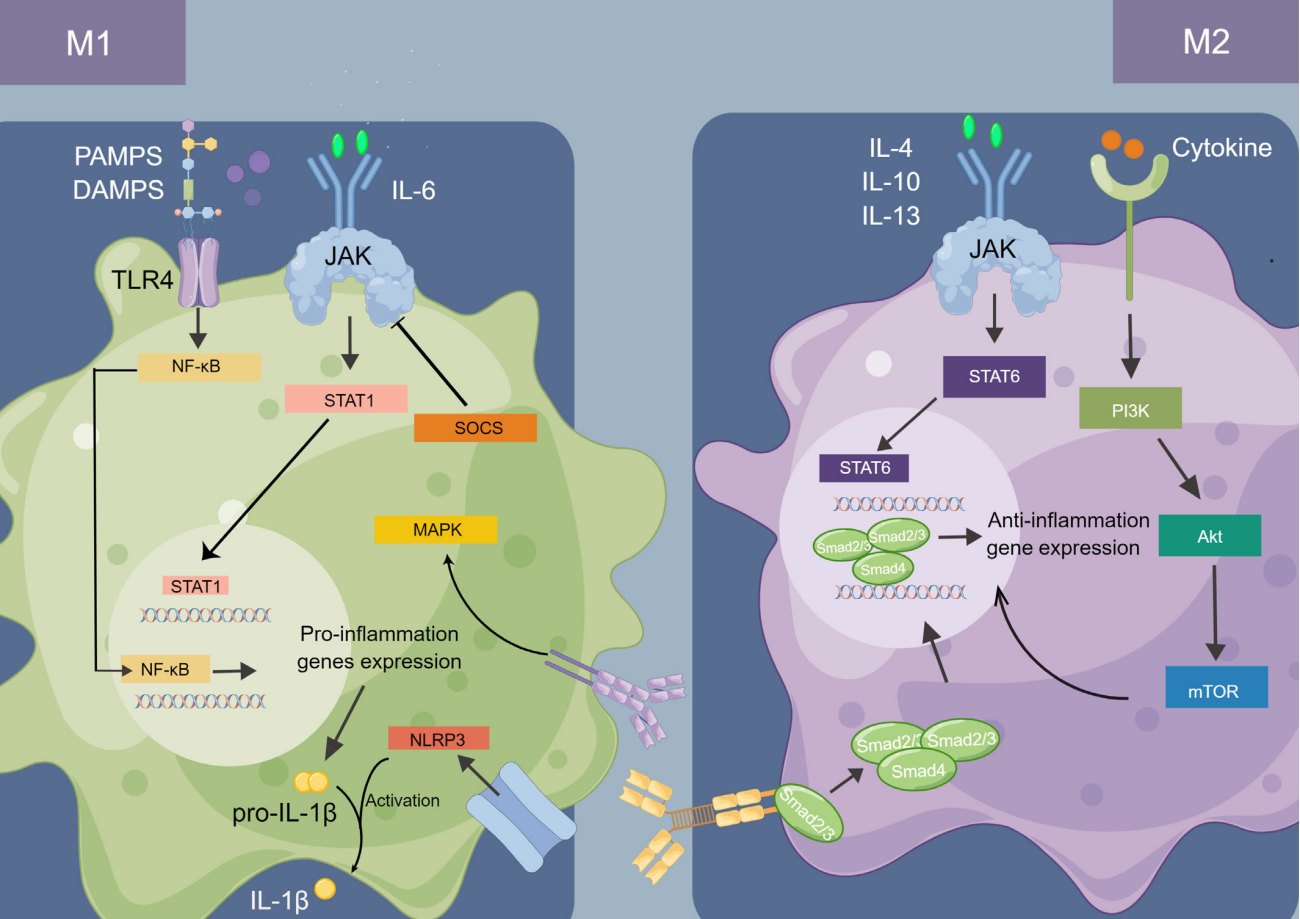
Signaling pathways in macrophage polarization.

Herein, we summarized several signaling pathways in macrophage reprogramming, including NF‐κB, MAPK, TGF‐β, JAK/STAT, PI3K/Akt/mTOR, and NLRP3 (Figure 2). More in‐depth studies of more specific molecular mechanisms in the macrophage polarization will likely help translate it from bench to clinic since signaling pathways have multiple effectors and regulators in our body, which leads to complex and dynamic processes in the body.

### NF‐κB Signaling Pathway

The NF‐κB is generally acknowledged as a family of ubiquitous transcription factors in a great range of cells. It is involved in the adaptive and innate immune response and the pathogenesis of inflammation‐related disease, cancer, and other diseases.^63^ It is well established that the NF‐κB family consists of five distinct proteins, including p50, p52, Rel A (p65), Rel B, and c‐Rel, which yield homodimers and heterodimers, thus affecting cells’ biological process.^64^

Generally, the NF‐κB activation has the classical and alternative pathways. The classical one responds to external stimulus and mediates the activation of p50, p65, and c‐Rel, whereas p52 and Rel‐B are activated in the alternative pathway.^65^, ^66^ Then, the phosphorylation of the inhibitor of NF‐κB (IκB) proteins by the IκB kinase (IKK) complex (IKKα, ΙΚΚβ, ΙΚΚγ) is followed by degradation of IκB and release of the NF‐κB dimers from inhibition, which activate the transcription of target genes.^67^, ^68^ In contrast, the alternate pathway depends on NF‐κB inducing kinase (NIK), resulting in the phosphorylation of p52, and finally RelB/p52 dimer translocates into nuclear toward specific gene activation.^66^, ^69^

Emerging evidence has shown the critical role of the NF‐κB pathway in M1 polarization and subsequently inflammatory cytokine release, such as IL‐1β, IL‐6 and TNF‐α.^70^, ^71^ In OA, when PAMPs from microorganisms or DAMPs from damaged tissue are released, the activation of Toll‐like receptors (TLRs) leads to the M1 polarization, which is regulated *via* the TLR/NF‐κB signaling pathway.^72^ This implies that the block of TLRs might be a potential target for not only M1 polarization but also a promising OA therapy. Berberine was found to inhibit M1 polarization through a block of the TLR4/MyD88/NF‐κB signaling pathway and attenuate inflammation in the early phase.^73^ Likewise, Mo *et al*. (2023) found that knockdown of pellino1 (Peli1) could not only inhibit M1 polarization through the TLR signaling pathway but also produce anti‐apoptotic effects.^74^ Moreover, Barreto *et al*. demonstrated that lumican co‐stimulation with lipopolysaccharide provided a pro‐inflammatory stimulus, upregulating macrophage polarization toward the M1‐like phenotype.^75^ Consistently, chondrocyte death was also significantly upregulated in a TLR4‐dependent manner, which is a feature of OA, often through apoptosis and autophagy mechanisms.^76^ Beyond TLRs, p65 could also serve as a target for M1 polarization through inhibition of the NF‐κB pathway. Li *et al*. (2023) described the role of PCAF in NF‐κB activation during M1 polarization with regulation p65 activation through its pivotal role in p65 acetylation.^77^ Additionally, peroxisome proliferator receptors (PPAR) comprise a family of nuclear hormone receptors, which can evoke anti‐inflammatory outcomes and promote M2 polarization.^78^ Previous studies have demonstrated that PPAR directly interacts with p65 and p50, which prevents them from binding to specific DNA sequences.^79^ However, the link between inhibition of NF‐κB and enhancement of M2 polarization remains obscure and needs further investigation.

### Transforming Growth Factor β Signaling Pathway

Transforming growth factor β (TGF‐β) and related growth factors are secreted pleiotropic factors that play critical roles in embryogenesis and adult tissue homeostasis by regulating cell proliferation, differentiation, migration, and death.^80^ The TGF‐β superfamily comprises 33 members, including TGF‐β, Nodal, Activin, and bone morphogenetic proteins (BMPs).^81^ Binding TGF‐β family proteins to heteromeric complexes composed of two type I and two type II receptors on the plasma membrane, the type I transmembrane kinase is phosphorylated and activated following the activation of specific type II receptors. Activated type I receptor initiates intracellular signaling through phosphorylating the C termini of specific SMAD proteins, R‐Smads, the intracellular signaling effectors.^82^, ^83^ This event activates the R‐Smads and enables the formation of heteromeric complexes between two R‐Smads (SMAD2 and/or SMAD3) and one common‐Smad (co‐Smad), Smad4, and then translocates into the nucleus to direct transcriptional response and exert the transcription of target genes.^84^, ^85^ In addition, the inhibitory Smads (I‐Smads), Smad6 and Smad7, antagonize the signaling mediated by R‐Smads and co‐Smad.^86^

An increasing number of studies have suggested that macrophage polarization is associated with the TGF‐β signaling pathway. Numerous studies describe the role of M2‐phenotype tumor‐associated macrophage (TAM) in distinct cancers’ progression.^87^ CD155^+^ TAMs show an M2 phenotype and promote colorectal cancer cell migration, invasion, and tumor growth, regulated by TGF‐β‐induced STAT3 activation‐mediated release of matrix metalloproteinases (MMP) 2 and MMP9 in CRC cells.^88^ Similarly, the pro‐tumor roles of M2 TAMs by TGF‐β signaling have been described in breast cancer.^89^ Strikingly, constitutive miR‐182 deficiency and conditional knockout in macrophages impair M2‐like TAMs and breast tumor development, while reconstitution of miR‐182‐expressing macrophages promotes tumor growth.

A similar phenomenon of M2 polarization with an elevated level of the TGF‐β pathway has been observed in other diseases, such as OA.^90^, ^91^, ^92^ In Dai *et al*., squid type II collagen immunomodulates M2 macrophage polarization to skew the local OA microenvironment toward a pro‐chondrogenic atmosphere, with elevation of TGF‐β1 and TGF‐β3, and promotes cartilage repair under inflammatory conditions.^90^ Moreover, previous studies showed that eucommia ulmoides polysaccharides promoted the expression of TGF‐β accompanied with decreased M1 polarization and accumulating M2 polarization.^91^ Further, Tissue Gene‐C (TG‐C), a novel cell and gene therapy, has been used in the treatment of OA, which caused increased levels of TGF‐β1 and IL‐10, induced the expression of arginase 1, a marker of M2 macrophages, and decreased the expression of CD86, a marker of M1 macrophages.^92^ This evidence suggests a strong link between TGF‐β and macrophage polarization. However, how TG‐C induces an M2 macrophage‐dominant microenvironment through TGF‐β signaling was not mentioned in this study.^92^

### Mitogen‐Activated Protein Kinase Signaling Pathway

Mitogen‐activated protein kinase (MAPK) signaling pathways play a crucial role in human diseases, including inflammation and cancer,^93^ which are cascade reactions of three kinases. Initially, the most upstream kinase (MAPKKK) receives various extracellular and intracellular signals and activates the middle kinase (MAPKK) through direct phosphorylation. Then, MAPKK exclusively phosphorylate and activate MAPK to make its substrates execute specific cell fate decisions.^94^, ^95^, ^96^ MAPK, including extracellular signal‐regulated kinases (ERKs), c‐JUN NH2‐terminus kinases (JNKs), and p38 MAPK, typically affect cell proliferation, differentiation, apoptosis, and metabolism, regulating the biological behavior of cells through various signaling pathways.^97^

Emerging studies highlight the vital role of MAPK signaling in macrophage polarization in a variety of diseases. Over the past few years, a series of studies examined the macrophage polarization in the cancer, mechanistically regulated by MAPK signaling. In 2023, Zheng *et al*. identified that FBXO38 promotes macrophage immunosuppressive function by upregulating the expression of M2‐like genes through MAPK signaling, which affects the development of cancer or colitis.^98^ Qiu *et al*. discuss the pro‐tumor role of exosome miR‐519a‐3p, which activates the MAPK/ERK pathway, thereby causing M2‐like polarization of macrophages, accelerating the gastric cancer liver metastasis by inducing angiogenesis and promoting intrahepatic premetastatic niche formation.^99^

The regulatory roles of MAPK signaling in macrophage polarization and cartilage degradation have also been demonstrated in the pathogenesis and progression of OA.^100^, ^101^ Wu *et al*. found that the increase of IL‐6 and TNF‐α production, which are released by M1 macrophages and further deteriorate synovial inflammation, is mediated *via* the ERK, p38, and JNK signaling pathways.^40^, ^102^ Further, a recent study has shown that inhibition of the phosphorylation of MAPK in macrophages attenuates the infiltration of pro‐inflammatory M1‐type macrophages and articular cartilage degeneration,^103^ which suggests that the MAPK pathway might serve as a promising target for OA treatment. More interestingly, the MAPK pathway might become involved in the complication of wear particles in total joint replacements. This is evident in the case of M1 polarization induced by orthopaedic implant materials,^104^ where pre‐treatment of macrophages with MAPK inhibitors not only prevented M1 polarization but also pro‐inflammatory gene expression. This finding provides the basis for further improving our understanding of the signaling cascades activated by wear particles and paves the way for new treatments in the future.

### JAK/STAT Signaling Pathway

The JAK/STAT pathway is one of the crucial nodes that demonstrates how extracellular communication works, thus leading to multiple biological responses. A range of cytokines are recognized as ligands that activate the JAK/STAT pathway, which consequently regulates the target gene expression, including interleukins, colony‐stimulating factors (CSFs), hormone‐like cytokines, and growth factors.^105^, ^106^ JAK is a family of non‐transmembrane tyrosine kinases, including JAK1, JAK2, JAK3, and Tyk2. As signals transmit *via* receptors, the receptor‐associated JAKs activate, mediate tyrosine phosphorylation of receptors, and subsequently recruit STATs for further activation, tyrosine phosphorylation, and dimerization. The STAT family consists of STAT1, STAT2, STAT3, STAT4, STAT5a, STAT5b, and STAT6, which are activated by different cytokines and show distinct biological effects. Thereafter, the dimers translocate to the nucleus and finally regulate transcription.^107^

A wealth of evidence has described that macrophage polarization regulates the proinflammation and anti‐inflammation via the JAK/STAT pathway *in vitro* and in many diseases, such as rheumatoid arthritis,^108^, ^109^ inflammatory bowel disease,^110^ and cancers.^111^ Previous studies have analyzed different cytokines that induce M1 or M2 polarization through distinct JAK/STAT pathways. To sum up, proinflammatory cytokines like IL‐6 activate the M1 phenotype through the JAK2/STAT3 pathway,^112^ whereas pro‐healing or anti‐inflammatory cytokines like IL‐4, IL‐10, and IL‐13 promote alternative macrophage polarization via the JAK/STAT pathway.^113^ IL‐10 was reported to act as a signal through JAK1/STAT3, which was responsible for higher negative feedback control of inflammation.^114^ In addition, both IL‐4 and IL‐13 were found to activate the JAK3‐STAT6 signaling inducing M2 polarization.^115^ Meanwhile, in Bhattacharjee et al., IL‐13 was found to utilize JAK2‐STAT3 and Tyk2‐STAT1/STAT6, whereas IL‐4 could only use JAK1‐STAT3/STAT6.^116^

Suppressors of cytokine signaling (SOCS) proteins are a family of negative regulators of the JAK/STAT pathway, including CIS, SOCS1, SOCS2, SOCS3, SOCS4, SOCS5, SOCS6, and SOCS7. They function as an impediment or inhibitor by binding to the phosphorylated JAKs and their associated receptors. Therefore, SOCS proteins could be a prospective target for controlling JAK/STAT signaling in diseases.^117^ Likewise, SOCS might also have an effect on macrophage polarization by regulating the JAK/STAT pathway. For instance, downregulated SOCS1 expression was found to activate the JAK1/STAT1 pathway, thereby promoting the polarization of macrophages into M1 type.^118^ Fascinatingly, an increasing body of literature supports the strong link between altered cellular metabolism and macrophage polarization as an adaptation for alternative cellular functions. Several studies have described how the JAK/STAT pathway was involved in this process.^119^, ^120^, ^121^ Creatine was found to reprogram macrophage polarization toward M2 phenotype by suppressing the JAK/STAT1 pathway, while supporting IL‐4/STAT6/activated arginase 1 expression by enhancing chromatin remodeling.^120^ These findings reveal the underlying mechanism of the connection between changes in metabolism and macrophage polarization.

During the pathogenesis of OA, the JAK/STAT pathway has participated in the inflammatory and immune responses, cartilage remodeling, and metabolism.^122^, ^123^ Macrophage polarization is acknowledged as a crucial mechanism for inflammation in OA, as described above. Hu *et al*.^124^ and Dai *et al*.^90^ reported STAT6‐regulated M2 polarization *in vitro* and *in vivo*, which could be a potential target for OA treatment. More studies on OA mechanisms and using JAK/STAT as a therapeutic target are urgently needed.

### PI3K/Akt/mTOR Signaling Pathway

The PI3K includes three classes of enzymes: class I, II, and III. Among them, class I PI3Ks attract wide concentration from researchers. Initiated by growth factors, cytokines, and insulin, PI3Ks are downstream molecules of tyrosine kinases, G protein‐coupled receptors and GTPases, which activate PI3Ks.^125^ Later, activated PI3Ks catalyze the phosphorylation of phosphatidylinositol‐4,5‐bisphosphate into phosphatidylinositol‐3,4,5‐trisphosphate, which is a second messenger and further activates downstream effectors like protein kinases (e.g., Akts). Akts, consisting of Akt1, Akt2 and Akt3, are recruited to the inner surface of cytoplasmic membrane, where it is phosphorylated by phosphoinositide‐dependent kinase‐1. Thereafter, it activates various downstream molecules, such as mammalian target of rapamycin (mTOR), thus engaging in the biological process, including cell proliferation, survival, metabolism, apoptosis, and motility.^126^

Several studies emphasize the significance of the PI3K/Akt/mTOR pathway in cancer, with its dysregulation in almost all human cancers, including breast cancer, colorectal cancer, and hematologic malignancies.^127^ In recent years, a growing number of studies have focused on the PI3K/Akt pathway in macrophage polarization during cancer progression. In Geraldo *et al*., SLIT2 promoted microglia tumor‐supportive polarization through ROBO1‐ and ROBO2‐mediated PI3K‐γ activation, regulating the glioblastoma microenvironment and immunotherapeutic target for brain tumors.^128^ A similar phenomenon has been observed in esophageal squamous cell carcinoma.^129^ Interestingly, FOXO1(+) tumor‐induced M2 macrophages promoted tumor proliferation through the FAK–PI3K–AKT pathway and could be impeded by the PI3K inhibitor. Beyond the cancer, the PI3K/Akt pathway mediated macrophage polarization also becomes involved in the pathogenesis of other diseases, such as rheumatoid disease,^130^ pancreatitis,^131^ and OA.^132^

A wealth of studies reveal the correlation between the PI3K/Akt/mTOR signaling pathway and OA pathogenesis, including versatile functions in cartilage, synovial tissue, and subchondral bone.^133^ Of note, previous studies have demonstrated that PI3K/Akt/mTOR engaged in the process of macrophage reprogramming in OA.^132^ Several studies have demonstrated that the PI3K/Akt/mTOR pathway is a critical step for M2 polarization. Liu *et al*.^134^ used 3D‐M‐EF scaffolds to induce M2 polarization through PI3K/Akt signaling *in vitro*, which provided a beneficial microenvironment for tissue regeneration and engineering. Similarly, Li *et al*.^135^ modulated the PI3K/Akt to alter macrophage to M2 phenotype through mRNA mediation in an OA model. In addition, PI3K/Akt signaling potentially regulated M1 macrophages to exert anti‐inflammatory roles. In 2023, metformin was found to hamper the M1 polarization of synovial sublining macrophages through the PI3K/Akt pathway, thus lessening cartilage loss and finally attenuating OA development.^136^ However, related findings have rarely been reported so far, which means further exploration of the underlying mechanism is needed.

### NLRP3 Pathway

NLRP3 is a critical Nod‐like receptor (NLR) family member that forms the inflammasomes, protein complexes following the recognition of PAMPs and DAMPs during infection or stress.^137^ Inflammasomes consist of a sensor receptor, the adaptor apoptosis‐associated speck‐like protein containing a CARD (ASC), and the effector protease caspase 1.^138^ Most inflammasome receptors are NLR sensors, an essential family of PRRs, including molecules like NLRP1, NLRP3, and NLRP6. Among them, NLRP3 is the most characterized inflammasome, which comprises a pyrin domain (PYD), a NACHT domain hydrolyzing nucleotides with ATPase activity, and a leucine‐rich repeat (LRR) domain.^139^ Generally, the classical activation of NLRP3 pathway occurs in two signal steps. The first one is a prime signal, which requires activation of TLRs and the NF‐κB pathway, thus increasing the pro‐IL‐1β, pro‐IL‐18, and NLRP3 protein in the cytosol.^140^ Then, triggered by a great range of PAMPs or DAMPs, the second signal induces NLRP3 oligomerization and recruits downstream molecules, ASC and procaspase‐1, to come together to form the inflammasome.^137^ Ultimately, the NLRP3 activates caspase‐1, which cleaves pro‐inflammatory cytokines into an active form, thereby leading to an immune response.^141^ Furthermore, active caspase‐1 also triggers the cleavage of inflammatory cytokines IL‐1β and IL‐18 and gasdermin D, which induces a proinflammatory programmed cell death known as pyroptosis.^142^ Additionally, dysregulation of NLRP3 inflammasome could drive a variety of inflammation responses associated with diseases such as OA.^143^

Emerging evidence has shown that NLRP3 might be one of the regulatory mechanisms of macrophage polarization in diseases. Several previous studies have supported this suggestion *in vivo* and *in vitro*. Wisitpongpun *et al*. (2022) found that oleamide promoted M1 macrophage polarization and increased IL‐1β production by activating the NLRP3 inflammasome in primary monocyte‐derived macrophages.^144^ Similarly, M1 macrophage polarization requiring NLRP3 inflammasome activation mediated Th1 and Th17 differentiation, thus leading to choline‐metabolized trimethylamine N‐oxide‐induced graft‐versus‐host disease. In addition, Liu *et al*. showed that ubiquitin‐specific protease 19 (USP19) acts as an anti‐inflammatory switch that inhibits inflammatory responses and promotes M2‐like macrophage polarization by stabilizing NLRP3 inflammasome *in vitro* and in a peritonitis model *in vivo*.^145^ Appealingly, similar results concerned critical role of NLRP3 inflammasome in macrophage polarization have been reported in OA, which provides a promising therapeutic strategy for OA. Sun *et al*. (2022) revealed that inhibition of transient receptor potential channel subfamily V member 4 (TRVP4), an ion channel, inhibited M1 macrophage polarization through the ROS/NLRP3 pathway, consequently attenuating OA progression.^146^ In the same manner, Luo *et al*. demonstrated that IL‐37 induces M2 macrophage polarization through a process that requires IL‐1R8/NLRP3, which appears to be a potential therapy target for temporomandibular joint OA.^147^

## Application of Macrophage Polarization in the Treatment of Osteoarthritis

As is well known, treatments for OA are increasingly diverse, but there is still a need for more effective ways to alleviate the inflammatory progression. There are many ways to treat OA, including physical stimulus, chemical compounds, and biological molecules. Studies have demonstrated that most of these methods are related to macrophage polarization. Thus, we summarize the treatment of OA by targeting macrophage polarization as a potential target (Table 1).

**TABLE 1:** Summary of information on osteoarthritis treatment associated with macrophage polarization.

Type	Name		Study sample	Mechanism; function	Signaling pathway; target	Efficacy	Reference
Physical Stimulus	Moderate physical activity		MIA‐induced OA rat model	MPA generates synovial fluid and lipids in the infrapatellar fat pad, producing LXA_4_ in the knee joint. LXA_4_ act on synovial macrophages to promote M2 polarization	–	M2↑	148
	Low intensity pulsed ultrasound		DMM‐induced OA mouse model; THP‐1 cells, RAW264.7 cells	LIPUS downregulates the high expression of LPS‐induced p‐JNK and p‐p65 proteins; inhibites NF‐κB nuclear translocation in osteoblasts induced by LPS	JNK; NF‐κB	M1↓, M2↑	149
Chemical Compounds	Hyaluronic acid		Human primary synoviocytes obtained from knee joint OA patients; THP‐1 cells	HMW‐HA can inhibit the expression of GRP78 and the activiation of NF‐κB	GRP78/NF‐κB; target: CD44 receptor	M1↓, M2↑	70
	Rapamycin		CIOA and DMM‐induced OA mouse model	Rapamycin inactivates the complexes of the mTOR. Activated mTORC1 increases M1 polarization in both CIOA and DMM models, while inhibited mTORC1 enhanced M2 polarization and alleviated CIOA in mice	Target: mTORC1	M1↓, M2↑	26
	Itaconate		DMM‐induced OA mouse model; RAW264.7 macrophages	Exogenous supplementation of itaconate can activate Nrf2, and accordingly inhibit the STING‐dependent NF‐κB pathway	Nrf2/STING/ NF‐κB	M1↓, M2↑	150
	Platelet‐Rich Plasma		CIOA‐induced OA mouse model	PRP contains high levels of IL‐1Ra that can inhibit acute inflammation caused by IL‐1	Target: IL‐IR	M2↑	151
	Single compounds derived from TCM	Fargesin	CIOA‐induced OA mouse model; RAW264.7 macrophages	M1 macrophages treated with fargesin reduced the expression of catabolic markers (MMP13, RUNX2, and ColX) and increases chondrogenic markers (Col2a1 and SOX9) in OA chondrocytes	p38/ERK MAPK p65/NF‐κB	M1↓, M2↑	152
		Frugoside	CIOA‐induced OA mouse model; RAW264.7 cells	Frugoside inhibits macrophage M1 polarization by partially downregulating miR‐155 expression	Target: miR‐155	M1↓	153
		Quercetin	OA mouse model induced by removing the medial meniscus and transecting the anterior meniscotibial ligament; RAW264.7 cells	Quercetin inhibites the expression of inflammatory mediators and matrix degrading proteases and upregulates expression of cartilage anabolic factors in IL‐1β‐induced rat chondrocytes	Akt/NF‐κB	M2↑	124
Biological Molecules	Mesenchy‐me stem cells and their exosomes	BMSC‐derived exosomes	OA mouse model induced by the modified Hulth technique; RAW264.7 cells	BMSC‐derived exosomes promote the transformation of synovial macrophages from M1 to M2, reducing the infiltration of synovial inflammatory cells	–	M1↓, M2↑	154
		hUCMSCs	ACLT‐induced OA rat model	hUCMSCs‐EVs could alleviate OA progression likely *via* transferring key proteins and miRNAs to regulate the PI3K‐Akt signaling pathway	PI3K‐Akt	M1↓, M2↑	135
		AMSCs	Synovial tissues obtained from OA patients; subcutaneous abdominal fat obtained from healthy patients; human monocytes	PGE2, produced by ASC, directly inhibits the inflammatory cytokines TNFα and IL‐6 and can induce production of the anti‐inflammatory cytokine IL‐10 and expression of CD163 and CD206	PGE2/COX2	M1↓, M2↑	155
		IPFP MSC‐derived exosomes	Infrapatellar fat pad and articular cartilage obtained from OA patients	Exosomes derived from mesenchymal stem cells in IPFP can inhibit the mTOR signaling pathway through miR‐100‐5p, enhance autophagy, inhibit apoptosis, promote extracellular matrix synthesis	mTOR target: miR‐100‐5p	M1↓	54
	Artificial M2 macrophage		Papain solution‐induced OA mouse model; RAW264.7 cells	AM2M could decrease joint surface erosion, chondrocyte apoptosis and loss of glycosaminoglycan content, and downregulate the secretion level of the inflammatory factors such as IL‐Iβ, IL‐6, and IL‐17	–	M2↑	156
	Meta‐Defensome		Patients with OA; RAW264.7 cells	Meta‐defensomes reprogram the mitochondrial metabolism of M1 macrophages by scavenging mitochondrial reactive oxygen species and inhibiting mitochondrial NO synthase, thereby increasing mitochondrial transcription factor A expression and restoring aerobic respiration.	–	M1↓, M2↑	157
	Modified ZIF‐8 Nanoparticles		ACLT‐induced OA rat model	Infiltration of the synovial M1 macrophages (CD16/32‐positive cells is decreased after treatment with modified NPs, and NPs upregulates synovial M2 phenotype macrophages (CD206‐positive cells)	–	M1↓, M2↑	158

Abbreviations: ACLT, anterior cruciate ligament transection; Akt, protein kinase B; AM2M, artificial M2 macrophage; AMSCs, adipose‐derived mesenchymal stem cells; BMSC, bone marrow mesenchymal stem cell; CIOA, collagenase‐induced OA; ColX, collagen X; Col2a, collagen Type II Alpha 1; COX2, cyclooxygenase‐2; DMM, destabilization of the medial meniscus; ERK, extracellular regulated protein kinase; EVs, extracellular vesicles; GRP78:78‐kD glucose‐regulated protein; HMW‐HA, high molecular weight hyaluronic acid; hUCMSCs, human umbilical cord mesenchymal stem cells; IL‐IR, interleukin‐1 receptor; IPFP‐MSC, infrapatellar fat pad mesenchymal stem cell; JNK, Jun N‐terminal kinase; LIPUS, low intensity pulsed ultrasound; LPS, lipopolysaccharide; LXA4, lipoxin A4; MAPK, mitogen‐activated protein kinase; MIA, monoiodoacetic acid; miR‐100‐5p, microRNA‐100‐5p; miR‐155, microRNA‐155; MMP13, matrix metalloproteinase protein 13; MPA, moderate physical activity; mTORC1, mammalian target of rapamycin C1; NF‐κB, nuclear factor kappa‐light‐chain‐enhancer of activated B cells; NPs, nanoparticles; Nrf2, nuclear factor‐erythroid 2‐related factor 2; OA, osteoarthritis; PGE2, prostaglandin E2; PI3K, phosphatidylinositol 3‐kinase; RUNX2, Runt‐related transcription factor 2; STING, stimulator of interferon genes; PRP, platelet rich plasma; TNFα, tumor necrosis factor alpha.

### Physical Stimulus Targeted at Macrophage Polarization

#### Moderate Physical Activity

Moderate physical activity (MPA) refers to activity that requires 3.0–5.9 times higher energy than that of the resting state and plays a crucial role in joint health.^159^, ^160^ In recent years, MPA has been widely used as a treatment in patients after arthroplasty due to increasing muscle strength, reducing postoperative pain, recovery times, and costs.^161^, ^162^ In a rat model with peroxisome proliferator‐activated receptors γ (PPARγ) deletion in myeloid cells, Silveira *et al*. found that PPARγ was essential to exercise‐induced M2 peritoneal macrophage recruitment and polarization, which strongly supported the association between exercise and macrophage polarization.^163^ There is also evidence showing that MPA produces a hypoxic environment in blood vessels, which promotes platelet aggregation, lipoxygenase activation, and LXA4 generation. In the above study, LXA4 has also been proven to promote M2 polarization and reduce inflammatory cytokines in the synovium, thereby relieving OA.^148^ In summary, MPA could facilitate M2 polarization of synovial macrophages, which is a potential therapeutic tool for OA. However, there are many forms of MPA. Additionally, treadmill exercise in animals cannot mimic the activities or common exercises that OA patients might perform. Further, participation in any physical activity must be balanced with the injury risk, and extreme fitness trends of the past result in more knee OA cases. As such, mitigating injury risk is paramount for participating in physical activity.^164^, ^165^

#### Low‐Intensity Pulsed Ultrasound

Low‐intensity pulsed ultrasound (LIPUS), a kind of non‐invasive and safe physical therapy, has been widely used in the treatment of fractures and other musculoskeletal diseases.^166^ A large number of clinical studies suggest that LIPUS can alleviate the degeneration of knee cartilage. Therefore, it is regarded as a potential treatment for OA.^167^ LIPUS was found to increase the specific protein CD163, one of the major changes in macrophages converted to the M2 subtype, when it was used at the later stage of tendon–bone interface (TBI) healing. This evidence demonstrated that macrophage polarization might be a potential mechanism of LIPUS treatment.^168^ Sun *et al*. (2020) also showed that LIPUS treatment decreases the proportion of M1 macrophages and increases the M2 macrophages in the joint synovium through suppressing the JNK and NF‐κB signaling pathways.^149^ Above all, LIPUS has been considered an effective strategy for patients with OA.

The main limitation of this therapy is that no clinical studies have been reported on its role in the treatment of OA patients. Scientific research is generally carried out on animals, whose knees have different anatomical and physiological characteristics to humans. On the one hand, this might affect the reproducibility of results obtained in human clinical studies in the future, and on the other hand, there is a lack of parameter and dosage information for giving this physical therapy to humans, which has implications for safety.^169^

### Chemical Compounds Targeted at Macrophage Polarization

#### Hyaluronic Acid

Intra‐articular injection of hyaluronic acid (HA) is a common treatment of knee OA for patients. Nonsteroidal anti‐inflammatory drugs are not effective or contraindicated to replace the lost HA in synovial fluid.^170^ Macrophage‐derived inflammatory and degenerative molecules, including TNF‐α, IL‐6, IL‐1β, MMPs, and ADAMTS, which lead to synovitis, have significantly reduced expression after using HA alone or in combination.^171^, ^172^, ^173^ Jin *et al*. (2022) showed that the ratio of M1 to M2 was steadily increased after HA intra‐articular injection, which means intra‐articular hyaluronic acid (IAHA) might play an anti‐inflammatory functional role through macrophage polarization.^173^ Furthermore, Lee *et al*. found that high molecular weight HA (HMW‐HA) could affect the polarization of synovial macrophages through the inhibition of the GRP78/NF‐κB pathway, which increased the M2/M1 ratio and decreased pro‐inflammatory cytokines.^70^ However, Rayahin *et al*. demonstrated that hyaluronic acid has molecular weight‐dependent effects in modulating the macrophage phenotype.^174^ HMW‐HA increases the M2 phenotype, whereas HA of a low molecular weight increases the M1 phenotype.^70^

However, until now, the effects of age, symptoms, and severity of OA patients on the clinical efficacy of HA have been unclear. Some studies have shown that older patients and those with advanced arthritis are less likely to benefit from intra‐articular HA injections.^175^ Other studies have shown that younger patients with moderate symptoms or only early radiological signs of OA do not appear to gain any benefit from HA injections.^176^ Therefore, long‐term follow up results after such injections remain to be investigated in future studies.

#### Rapamycin

Rapamycin, a potential inhibitor of T cell proliferation, is widely used as a medication to prevent organ transplant rejection through inactivating the mTOR. The mTOR, a ubiquitous serine/threonine kinase, plays a crucial role in regulating cell growth, proliferation, and survival.^177^ There is increasing evidence that the mTOR is closely related to macrophage polarization.^178^, ^179^, ^180^, ^181^, ^182^ There are two interacting complexes of the mTOR, mTORC1 and mTORC2, which regulate T cell lineage specification and macrophage differentiation.^177^ The present study demonstrates that treatment with rapamycin *in vitro* reduces mTORC1 and enhances mTORC2 activity in T cells of healthy individuals. However, whether long‐term treatment with rapamycin can also activate mTORC2 *in vivo* is unclear. Therefore, it is uncertain whether the therapeutic effect of rapamycin in OA patients stems solely from the blocking of mTORC1 or also involves the activation of mTORC2.^177^ As mentioned above, Zhang *et al*. found that enhancement of M1 macrophages was associated with the mTORC1 activation in the synovium of OA patients.^26^ They also concluded that mTORC1‐induced M1 polarization stimulates inflammatory cytokine production and Rspo2 secretion, which promotes a series of characteristic changes in OA, such as degradation of matrix proteoglycan and cartilage.^26^

#### Itaconate

Itaconate, regulated by immune‐responsive gene 1 (Irg1), is an unsaturated dicarboxylic acid derived from the tricarboxylic acid cycle.^183^ A previous study has shown that itaconate can be highly expressed in macrophages to inhibit their inflammatory activities, thus playing a role in the regulation of macrophage function.^184^ There is mounting evidence demonstrating that itaconate can reduce the degree of inflammation as an Nrf2 agonist.^183^, ^184^, ^185^, ^186^, ^187^

Itaconate was found to have the ability to alleviate the progression of OA by reducing the destruction of cartilage and regulating macrophage polarization. Pan *et al*. found that four‐octyl itaconate (4‐OI) could improve autophagy in chondrocytes by regulating the PI3K/AKT/mTOR signaling pathway.^188^ Interestingly, Guo *et al*. (2022) also demonstrated that itaconate could promote M2 polarization in macrophages, reducing the release of pro‐inflammatory cytokines such as IL‐1β, IL‐6, and TNF‐α, thereby relieving synovial inflammation and chondrocyte apoptosis.^150^

Nevertheless, most of the current studies have only investigated the effects of itaconate on the inflammation and injury of articular cartilage in the Nrf2 pathway.^189^, ^190^ Therefore, more experiments should be performed in future to reveal the signaling pathway of itaconate in chondrocyte protection.

#### Platelet‐Rich Plasma

Platelet‐rich plasma (PRP) is a platelet concentrate obtained from autologous whole blood after centrifugation that contains a large number of growth factors such as PDGF, transforming growth factor β (TGF‐β), insulin‐like growth factor 1 (IGF‐1), and vascular endothelial growth factor (VEGF).^191^ PRP releases various growth factors (mentioned above) that are involved in the regulation of bone regeneration in the local microenvironment of bone defects to accelerate wound healing and the tissue repair process.^192^ The result of a previous study that PRP from OA patients could contribute to the inhibition of chondrocyte matrix synthesis and enhanced macrophage inflammation *in vitro* demonstrated the strong link between PRP and OA treatment.^193^

Of note, data from a mouse OA model suggested that the articular cavity of knees might have fewer proinflammatory and more anti‐inflammatory macrophages after injection of PRP. Surprisingly, Khatab *et al*. demonstrated that PRP suppresses IL‐1‐induced acute inflammation and promotes M2 macrophage polarization due to the high level of IL‐1 receptor antagonist it contains.^151^ Furthermore, Uchiyama *et al*. proved that injection of PRP in knees could transform M1 macrophages into M2 macrophages in different ways, such as directly polarizing M1 macrophages to M2 macrophages, aggregating macrophages and monocytes around the joint, and unifing polarizing into M2 macrophages.^194^ These results demonstrate that PRP is a therapeutic target of OA through its function on macrophage polarization in the joint and synovium.

Overall, most of the low‐quality research evidence suggests that intra‐articular injection of PRP is a safe treatment with the potential to provide symptomatic benefits for OA in the short term, and there are studies suggesting that younger patients and those with less structural changes in the knee joint might be more sensitive to PRP.^195^ However, these results need to be confirmed in high‐quality clinical trials, and further research is needed to identify patient characteristics that make them suitable for PRP. Due to the lack of research in this area, there is currently no recommendation on the optimal PRP protocol for patients with OA.

#### Single Compounds Derived from Traditional Chinese Medicine

The formulations used in traditional Chinese medicine (TCM) are very intricate and include a wide range of effective substances.^196^ It is crucial to separate the components and purify them to create powerful single compounds that can be used to increase medicine efficacy, lessen adverse effects, and study the pharmacological process. As a result, numerous researchers have investigated and analyzed tiny compounds derived from TCM to prevent inflammation and degeneration against OA.^197^, ^198^, ^199^, ^200^

Moreover, in terms of macrophage polarization, several studies have focused on the active ingredients of TCM herbs targeted at macrophage reprogramming for the treatment of OA. Fargesin, which is one of the main components of *Magnolia fargesii*, has traditionally been used to treat sinusitis and inflammation.^201^ According to a recent study, fargesin could promote the phenotypic transformation of macrophages from M1 to M2 and partially prevent cartilage degeneration by downregulating p38/ERK MAPK and p65/NF‐κB signaling.^152^ In addition, a number of extractions, including frugoside from *Calotropis gigante* and quercetin, a naturally occurring flavonoid present in a large variety of fruits and vegetables, have been shown to retard the degradation of cartilage and extracellular matrix as well as chondrocyte hypertrophy by inhibiting M1 polarization or inducing M2 polarization.^124^, ^153^ Nevertheless, the translation of herbal substances into clinics has a number of challenges, including potential liver and kidney toxicity, the difficulty of standardization, and the low stability or solubility of tiny molecules in serum or synovial fluid.^196^, ^202^ The efficacy of TCM herbs for the treatment of OA patients cannot be adequately evaluated.^203^ Therefore, sufficient representative animal models and preclinical experiments are required for further exploration.

### Biomaterial‐Based Applications Targeted at Macrophage Polarization

Biomaterials of both synthetic and natural origin have been investigated in the context of tissue regeneration.^204^ When exposed to synthetic biomaterials, macrophages are polarized toward anti‐inflammatory functions as opposed to their normal pro‐inflammatory reaction to natural biomaterials, such as the dermis of mammalian origin.^205^, ^206^ Further, a number of variables, such as the surface morphology, design, and chemical composition, will have an effect on the *in vivo* and *in vitro* behavior of macrophages, which will further influence the development of tissue regeneration.^204^

#### Mesenchyme Stem Cells and their Exosomes

**FIGURE 3:**
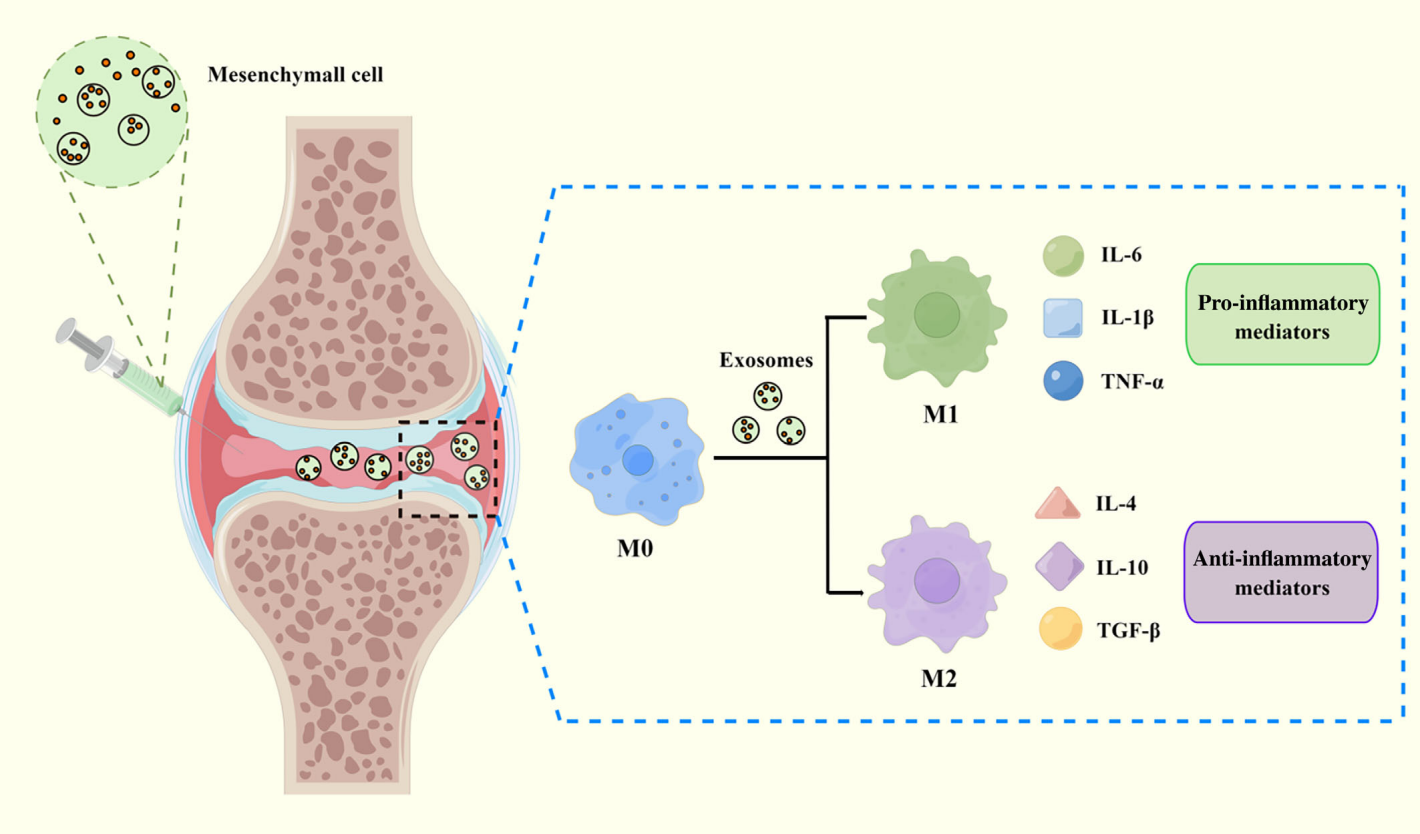
Mesenchyme stem cells and their exosomes as a treatment for osteoarthritis (OA).

Due to their chondrogenic potential and immunomodulatory capacities, mesenchyme stem cells (MSCs) are being studied extensively as a potential therapy for OA.^207^ A growing number of studies have come to the conclusion that the paracrine mechanism is the principal role of MSCs, and exosomes generated from MSCs serve as an important medium to exert MSCs’ therapeutic effects.^208^ MSC‐derived exosomes can also influence macrophages, leading them to become more polarized into M2 macrophages and produce more anti‐inflammatory cytokines.^209^ Zhang *et al*. found that exosomes derived from bone mesenchyme stem cells might delay the progression of OA by promoting a switch of macrophages from M1 to M2 phenotype.^154^ A similar phenomenon was observed by Cosenza *et al*. through a mouse OA model induced by intra‐articular injection collagenase.^210^ In addition to bone marrow, extracellular vesicles from other origins, including infrapatellar fat pads,^54^ human umbilical cord,^135^ and adipose tissue,^155^ have been demonstrated to influence macrophage polarization and relieve OA. Notably, although diverse exosomes generated by different tissues have been demonstrated to effectively prevent OA progression through altering the macrophage phenotype, the precise nature of their effects is not fully understood, particularly the specific role of miRNA and proteins in these microparticles.^135^ Therefore, further thorough studies are needed to identify the crucial internal contents of exosomes and specific molecular mechanisms on OA (Figure 3).

#### Artificial M2 Macrophages

Current data suggest that M1 phenotype macrophages are mainly involved in harmful and crippling chondrocyte conditions, whereas the M2 phenotype can aid in cartilage restoration. As a result, macrophage M2 polarization has significant promise for treating OA, albeit with low expression of some pro‐inflammatory cytokines that can cause cartilage damage.^211^, ^212^, ^213^ To regulate the beneficial secretion of M2 macrophages, an artificial M2 macrophage, mainly consisting of macrophage membrane as the “shell” and inflammation‐responsive nanogel as the “yolk,” was proposed and fabricated by Ma *et al*. to enhance the therapeutic efficacy of M2 macrophages in the treatment of OA.^156^ They demonstrated that this artificial M2 macrophage could break the severe circle of OA owing to its prominent characteristics, including its ability to target inflammation, the customizable rate of medication release in response to OA activity, and the prolonged retention duration in the joint cavity. Transplantation of exogenous M2 macrophages could be considered for treatment of OA, with the major challenge being high adaptability and plasticity in the microenvironment, which might lead to the loss of macrophage phenotype. Therefore, Liang *et al*. (2022) created a stable exogenous macrophage model by knocking out tumor necrosis factor receptor 1 (TNFR1) and using Cas9‐ribonuclear protein (Cas9‐RNP) complexes and electroporation to overexpress IL‐4, thus locking the interstitial synovial macrophage in the M2a phenotype (L‐M2a).^214^ Similarly, the modified L‐M2a macrophages retained a superior anti‐inflammatory and pro‐regenerative capacity in the inflammatory OA microenvironment, thus representing an ideal new strategy for the disease‐modifying therapy of OA.

#### Metabolic Reprogramming Compounds

Currently, several studies on the treatment of OA have focused on mitochondrial metabolism and how it relates to M1/M2 imbalance.^157^, ^158^ It has been reported that oxidative stress and mitochondrial dysfunction both play an important role in the dysregulation of the inflammatory response.^215^, ^216^ The process of oxidative phosphorylation is decreased by the activation of inflammatory M1 macrophages, which prevents their repolarization.^217^ Moreover, in disorders associated with inflammation, mitochondrial reactive oxygen species (mtROS) is essential for driving macrophage polarization to the inflammatory M1 phenotype.^218^, ^219^ Consequently, improving the reprogramming of inflammatory M1 macrophages into anti‐inflammatory M2 macrophages to control the disease might be possible by therapeutically restoring mitochondrial activity. In line with this, Zhang *et al*. (2022) have developed a novel meta‐defensome by metabolically engineering PLGA nanoparticles (MMP) enclosed in macrophage membrane to target M1 macrophages for selective cargo release in mitochondria.^157^ *In vivo*, in a collagenase‐induced OA mice model, the OA synovium was successfully defended by the meta‐defensome against inflammatory stress, and cartilage was shielded from deterioration by scavenging mtROS, restoring aerobic respiration and successfully repolarizing the M2 phenotype. In addition, the modified zeolitic imidazolate framework‐8 (ZIF‐8) nanoparticles synthesized by Zhou *et al*. was demonstrated to effectively catalyze H_2_O_2_ to produce O_2_ and eliminate NO, hence inhibiting hypoxia‐inducible factor 1α and further rescuing mitochondrial function. More importantly, through this metabolic reprogramming pathway, these modified nanoparticles upregulated M2 macrophage infiltration in the synovium, further inhibiting cartilage degeneration. Therefore, a metabolic reprogramming strategy targeted at mitochondria might pave the way for new OA therapy.^158^

## Future Directions

Macrophage polarization, as first stated by Mills *et al*.,^220^ is a kind of classification for the description of macrophage phenotypical and functional diversity in physiological and disease states, which is mainly based on the arginine metabolism. For decades, a growing literature has demonstrated the limitations of this concept, as the binary classifications could be an oversimplification, and there is a continuum of macrophage phenotypes between M1 and M2 macrophages *in vivo*.^221^, ^222^ However, studies continue to utilize M1 or M2 markers in the classification of macrophages because there is extensive experience in reprogramming macrophages based on their polarization, which is certainly an attractive strategy in many diseases.

Macrophages have functional plasticity, and when stimulated in the microenvironment of a specific tissue, they switch from one functional phenotype to another, which can be used as a target for disease treatment. For OA, applying this property to therapy requires understanding the microenvironment of OA, as well as the factors within it that might influence plasticity. Studies have shown that in addition to the M1 and M2 types of macrophages, M2 can also be divided into four subtypes with different functions, all with different cell expression markers.^223^ Therefore, studying the expression of macrophage surface markers and the production of specific biological factors at different stages of OA can be helful in determining disease progression.

In recent years, the rapid progress in single‐cell RNA sequencing, spatial transcriptomics, fate‐mapping, and multiplexed immunohistochemical technologies has revealed the unexpected diversity in macrophages. Indeed, several studies have demonstrated the synovial macrophage diversity during the pathogenesis in the OA,^224^, ^225^, ^226^ and in‐depth research focusing on the distinct functions, origins, and disease kinetics of macrophage subtypes would be indispensable for targeting these versatile cells.

## Conclusion

Osteoarthritis, as a prevalent chronic degenerative joint disease in middle‐aged and elderly individuals, is significantly influenced by the behavior of macrophages within joint tissues. Macrophage polarization, where these cells adapt to pro‐inflammatory or anti‐inflammatory states in response to environmental stimuli, plays a crucial role in the pathogenesis and progression of OA. This review's focus on synovial macrophages, adipose tissue macrophages, and osteoclasts reveals their substantial roles in OA development. Key findings in the field have highlighted various signaling pathways, such as NF‐κB, MAPK, and TGF‐β, involved in macrophage polarization related to OA. The potential for reprogramming macrophages based on these targets has emerged as a promising avenue for enhancing cartilage repair and mitigating OA progression.

## Author Contributions

YZ and XY conceived the concept of the manuscript. ZMY was responsible for the initial manuscript writing. DCJ, MZY and JT revised the manuscript. All authors participated the manuscript writing and approved the final manuscript.

## Conflict of Interest Statement

All authors have declared that there is no potential conflict of interest.

## Funding Informations

This study was support by the Department of Science and Technology of Sichuan Province (23ZDYF2641), Health Commission of Sichuan Province (2023‐118), Department of Science and Technology of Chengdu (2023‐GH02‐00075‐HZ), and National key research and development program of the Ministry of Science and Technology (2023YFB4606700).

## Ethics Statement

The data utilized for this study were derived from databases that are publicly accessible and do not contain any personally identifiable information. Consequently, ethical parameters are not applicable.

## References

[1] Katz JN , Arant KR , Loeser RF . Diagnosis and treatment of hip and knee osteoarthritis: a review. Jama. 2021;325(6):568–578. 33560326 10.1001/jama.2020.22171 PMC8225295

[2] Martel‐Pelletier J , Barr AJ , Cicuttini FM , Conaghan PG , Cooper C , Goldring MB , et al. Osteoarthritis. Nat Rev Dis Primers. 2016;2:16072. 27734845 10.1038/nrdp.2016.72

[3] Allen KD , Thoma LM , Golightly YM . Epidemiology of osteoarthritis. Osteoarthr Cartil. 2022;30(2):184–195. 10.1016/j.joca.2021.04.020 PMC10735233 34534661

[4] Tsezou A . Osteoarthritis year in review 2014: genetics and genomics. Osteoarthr Cartil. 2014;22(12):2017–2024. 10.1016/j.joca.2014.07.024 25456297

[5] Qiao L , Li Y , Sun S . Insulin exacerbates inflammation in fibroblast‐like Synoviocytes. Inflammation. 2020;43(3):916–936. 31981062 10.1007/s10753-020-01178-0 PMC7280329

[6] Pan L , Xie W , Fu X , Lu W , Jin H , Lai J , et al. Inflammation and sarcopenia: a focus on circulating inflammatory cytokines. Exp Gerontol. 2021;154:111544. 34478826 10.1016/j.exger.2021.111544

[7] Nedunchezhiyan U , Varughese I , Sun ARJ , Wu X , Crawford R , Prasadam I . Obesity, inflammation, and immune system in osteoarthritis. Front Immunol. 2022;13:907750. 35860250 10.3389/fimmu.2022.907750 PMC9289681

[8] Peshkova M , Lychagin A , Lipina M , di Matteo B , Anzillotti G , Ronzoni F , et al. Gender‐related aspects in osteoarthritis development and progression: a review. Int J Mol Sci. 2022;23(5):2767. 35269906 10.3390/ijms23052767 PMC8911252

[9] Maly MR , Marriott KA , Chopp‐Hurley JN . Osteoarthritis year in review 2019: rehabilitation and outcomes. Osteoarthr Cartil. 2020;28(3):249–266. 10.1016/j.joca.2019.11.008 31877379

[10] Driban JB , Harkey MS , Liu SH , Salzler M , McAlindon TE . Osteoarthritis and aging: young adults with osteoarthritis. Curr Epidemiol Rep. 2020;7(1):9–15.

[11] Tardito S , Martinelli G , Soldano S , Paolino S , Pacini G , Patane M , et al. Macrophage M1/M2 polarization and rheumatoid arthritis: a systematic review. Autoimmun Rev. 2019;18(11):102397. 31520798 10.1016/j.autrev.2019.102397

[12] Jiao Y , Zhang T , Zhang C , Ji H , Tong X , Xia R , et al. Exosomal miR‐30d‐5p of neutrophils induces M1 macrophage polarization and primes macrophage pyroptosis in sepsis‐related acute lung injury. Crit Care. 2021;25(1):356. 34641966 10.1186/s13054-021-03775-3 PMC8507252

[13] Wang C , Ma C , Gong L , Guo Y , Fu K , Zhang Y , et al. Macrophage polarization and its role in liver disease. Front Immunol. 2021;12:803037. 34970275 10.3389/fimmu.2021.803037 PMC8712501

[14] Boutilier AJ , Elsawa SF . Macrophage polarization states in the tumor microenvironment. Int J Mol Sci. 2021;22(13):6995. 34209703 10.3390/ijms22136995 PMC8268869

[15] Ginhoux F , Greter M , Leboeuf M , Nandi S , See P , Gokhan S , et al. Fate mapping analysis reveals that adult microglia derive from primitive macrophages. Science. 2010;330(6005):841–845. 20966214 10.1126/science.1194637 PMC3719181

[16] Hashimoto D , Chow A , Noizat C , Teo P , Beasley MB , Leboeuf M , et al. Tissue‐resident macrophages self‐maintain locally throughout adult life with minimal contribution from circulating monocytes. Immunity. 2013;38(4):792–804. 23601688 10.1016/j.immuni.2013.04.004 PMC3853406

[17] Schulz C , Perdiguero EG , Chorro L , Szabo‐Rogers H , Cagnard N , Kierdorf K , et al. A lineage of myeloid cells independent of Myb and hematopoietic stem cells. Science. 2012;336(6077):86–90. 22442384 10.1126/science.1219179

[18] Lavin Y , Mortha A , Rahman A , Merad M . Regulation of macrophage development and function in peripheral tissues. Nat Rev Immunol. 2015;15(12):731–744. 26603899 10.1038/nri3920 PMC4706379

[19] Culemann S , Grüneboom A , Nicolás‐Ávila JÁ , Weidner D , Lämmle KF , Rothe T , et al. Locally renewing resident synovial macrophages provide a protective barrier for the joint. Nature. 2019;572(7771):670–675. 31391580 10.1038/s41586-019-1471-1 PMC6805223

[20] Udalova IA , Mantovani A , Feldmann M . Macrophage heterogeneity in the context of rheumatoid arthritis. Nat Rev Rheumatol. 2016;12(8):472–485. 27383913 10.1038/nrrheum.2016.91

[21] Kraus VB , McDaniel G , Huebner JL , Stabler TV , Pieper CF , Shipes SW , et al. Direct in vivo evidence of activated macrophages in human osteoarthritis. Osteoarthr Cartil. 2016;24(9):1613–1621. 10.1016/j.joca.2016.04.010 PMC4992586 27084348

[22] Wood MJ , Leckenby A , Reynolds G , Spiering R , Pratt AG , Rankin KS , et al. Macrophage proliferation distinguishes 2 subgroups of knee osteoarthritis patients. JCI Insight. 2019;4(2):e125325. 30674730 10.1172/jci.insight.125325 PMC6413777

[23] Krausgruber T , Blazek K , Smallie T , Alzabin S , Lockstone H , Sahgal N , et al. IRF5 promotes inflammatory macrophage polarization and TH1‐TH17 responses. Nat Immunol. 2011;12(3):231–238. 21240265 10.1038/ni.1990

[24] Huang Z , Kraus VB . Does lipopolysaccharide‐mediated inflammation have a role in OA? Nat Rev Rheumatol. 2016;12(2):123–129. 26656661 10.1038/nrrheum.2015.158 PMC4930555

[25] Murray PJ , Allen JE , Biswas SK , Fisher EA , Gilroy DW , Goerdt S , et al. Macrophage activation and polarization: nomenclature and experimental guidelines. Immunity. 2014;41(1):14–20. 25035950 10.1016/j.immuni.2014.06.008 PMC4123412

[26] Zhang H , Lin C , Zeng C , Wang Z , Wang H , Lu J , et al. Synovial macrophage M1 polarisation exacerbates experimental osteoarthritis partially through R‐spondin‐2. Ann Rheum Dis. 2018;77(10):1524–1534. 29991473 10.1136/annrheumdis-2018-213450

[27] Fahy N , de Vries‐van Melle ML , Lehmann J , Wei W , Grotenhuis N , Farrell E , et al. Human osteoarthritic synovium impacts chondrogenic differentiation of mesenchymal stem cells via macrophage polarisation state. Osteoarthr Cartil. 2014;22(8):1167–1175. 10.1016/j.joca.2014.05.021 24911520

[28] Haltmayer E , Ribitsch I , Gabner S , Rosser J , Gueltekin S , Peham J , et al. Co‐culture of osteochondral explants and synovial membrane as in vitro model for osteoarthritis. PloS One. 2019;14(4):e0214709. 30939166 10.1371/journal.pone.0214709 PMC6445514

[29] Manferdini C , Paolella F , Gabusi E , Silvestri Y , Gambari L , Cattini L , et al. From osteoarthritic synovium to synovial‐derived cells characterization: synovial macrophages are key effector cells. Arthritis Res Ther. 2016;18:83. 27044395 10.1186/s13075-016-0983-4 PMC4820904

[30] Sun AR , Wu X , Liu B , Chen Y , Armitage CW , Kollipara A , et al. Pro‐resolving lipid mediator ameliorates obesity induced osteoarthritis by regulating synovial macrophage polarisation. Sci Rep. 2019;9(1):426. 30674985 10.1038/s41598-018-36909-9 PMC6344566

[31] Sun AR , Panchal SK , Friis T , Sekar S , Crawford R , Brown L , et al. Obesity‐associated metabolic syndrome spontaneously induces infiltration of pro‐inflammatory macrophage in synovium and promotes osteoarthritis. PloS One. 2017;12(8):e0183693. 28859108 10.1371/journal.pone.0183693 PMC5578643

[32] Wu CL , McNeill J , Goon K , Little D , Kimmerling K , Huebner J , et al. Conditional macrophage depletion increases inflammation and does not inhibit the development of osteoarthritis in obese macrophage Fas‐induced apoptosis‐transgenic mice. Arthritis Rheumatol. 2017;69(9):1772–1783. 28544542 10.1002/art.40161 PMC5611814

[33] Li L , Li Z , Li Y , Hu X , Zhang Y , Fan P . Profiling of inflammatory mediators in the synovial fluid related to pain in knee osteoarthritis. BMC Musculoskelet Disord. 2020;21(1):99. 32059658 10.1186/s12891-020-3120-0 PMC7023718

[34] Franzoni G , Mura L , Razzuoli E , de Ciucis CG , Fruscione F , Dell'Anno F , et al. Heterogeneity of phenotypic and functional changes to porcine monocyte‐derived macrophages triggered by diverse polarizing factors in vitro. Int J Mol Sci. 2023;24(5):4308. 36902099 10.3390/ijms24054671 PMC10003195

[35] Shapouri‐Moghaddam A , Mohammadian S , Vazini H , Taghadosi M , Esmaeili SA , Mardani F , et al. Macrophage plasticity, polarization, and function in health and disease. J Cell Physiol. 2018;233(9):6425–6440. 10.1002/jcp.26429 PMC13482560 29319160

[36] Zheng L , Wang Y , Qiu P , Xia C , Fang Y , Mei S , et al. Primary chondrocyte exosomes mediate osteoarthritis progression by regulating mitochondrion and immune reactivity. Nanomedicine (Lond). 2019;14(24):3193–3212. 31855117 10.2217/nnm-2018-0498

[37] Bailey KN , Furman BD , Zeitlin J , Kimmerling KA , Wu CL , Guilak F , et al. Intra‐articular depletion of macrophages increases acute synovitis and alters macrophage polarity in the injured mouse knee. Osteoarthr Cartil. 2020;28(5):626–638. 10.1016/j.joca.2020.01.015 PMC8963860 32044353

[38] Dakin SG , Coles M , Sherlock JP , Powrie F , Carr AJ , Buckley CD . Pathogenic stromal cells as therapeutic targets in joint inflammation. Nat Rev Rheumatol. 2018;14(12):714–726. 30420750 10.1038/s41584-018-0112-7

[39] Sun Z , Liu Q , Lv Z , Li J , Xu X , Sun H , et al. Targeting macrophagic SHP2 for ameliorating osteoarthritis *via* TLR signaling. Acta Pharm Sin B. 2022;12(7):3073–3084. 35865095 10.1016/j.apsb.2022.02.010 PMC9293663

[40] Bondeson J , Blom AB , Wainwright S , Hughes C , Caterson B , van den Berg WB . The role of synovial macrophages and macrophage‐produced mediators in driving inflammatory and destructive responses in osteoarthritis. Arthritis Rheum. 2010;62(3):647–657. 20187160 10.1002/art.27290

[41] Blom AB , van Lent PL , Libregts S , Holthuysen AE , van der Kraan PM , van Rooijen N , et al. Crucial role of macrophages in matrix metalloproteinase‐mediated cartilage destruction during experimental osteoarthritis: involvement of matrix metalloproteinase 3. Arthritis Rheum. 2007;56(1):147–157. 17195217 10.1002/art.22337

[42] Wang D , Chai XQ , Hu SS , Pan F . Joint synovial macrophages as a potential target for intra‐articular treatment of osteoarthritis‐related pain. Osteoarthr Cartil. 2022;30(3):406–415. 10.1016/j.joca.2021.11.014 34861384

[43] Takano S , Uchida K , Miyagi M , Inoue G , Fujimaki H , Aikawa J , et al. Nerve growth factor regulation by TNF‐α and IL‐1β in synovial macrophages and fibroblasts in osteoarthritic mice. J Immunol Res. 2016;2016:5706359. 27635406 10.1155/2016/5706359 PMC5007361

[44] Kriegova E , Manukyan G , Mikulkova Z , Gabcova G , Kudelka M , Gajdos P , et al. Gender‐related differences observed among immune cells in synovial fluid in knee osteoarthritis. Osteoarthr Cartil. 2018;26(9):1247–1256. 10.1016/j.joca.2018.04.016 29753948

[45] Kulkarni P , Srivastava V , Tootsi K , Electricwala A , Kharat A , Bhonde R , et al. Synovial fluid in knee osteoarthritis extends proinflammatory niche for macrophage polarization. Cell. 2022;11(24):4115. 10.3390/cells11244115 PMC9776803 36552878

[46] Clockaerts S , Bastiaansen‐Jenniskens YM , Runhaar J , van Osch GJVM , van Offel JF , Verhaar JAN , et al. The infrapatellar fat pad should be considered as an active osteoarthritic joint tissue: a narrative review. Osteoarthr Cartil. 2010;18(7):876–882. 10.1016/j.joca.2010.03.014 20417297

[47] Kouroupis D , Kaplan LD , Best TM . Human infrapatellar fat pad mesenchymal stem cells show immunomodulatory exosomal signatures. Sci Rep. 2022;12(1):3609. 35246587 10.1038/s41598-022-07569-7 PMC8897449

[48] Favero M , el‐Hadi H , Belluzzi E , Granzotto M , Porzionato A , Sarasin G , et al. Infrapatellar fat pad features in osteoarthritis: a histopathological and molecular study. Rheumatology (Oxford). 2017;56(10):1784–1793. 28957567 10.1093/rheumatology/kex287

[49] Barboza E , Hudson J , Chang WP , Kovats S , Towner RA , Silasi‐Mansat R , et al. Profibrotic infrapatellar fat pad remodeling without M1 macrophage polarization precedes knee osteoarthritis in mice with diet‐induced obesity. Arthritis Rheumatol. 2017;69(6):1221–1232. 28141918 10.1002/art.40056 PMC5449220

[50] Davis JE , Ward RJ , MacKay JW , Lu B , Price LL , McAlindon TE , et al. Effusion‐synovitis and infrapatellar fat pad signal intensity alteration differentiate accelerated knee osteoarthritis. Rheumatology (Oxford). 2019;58(3):418–426. 30346594 10.1093/rheumatology/key305 PMC6381765

[51] Wei W , Rudjito R , Fahy N , Bos KP , Verhaar JA , Clockaerts S , et al. The infrapatellar fat pad from diseased joints inhibits chondrogenesis of mesenchymal stem cells. Eur Cell Mater. 2015;30:303–314. 26629970 10.22203/ecm.v030a21

[52] Da‐Wa ZX , Jun M , Chao‐Zheng L , Sen‐Lin Y , Chuan L , De‐Chun L , et al. Exosomes Derived from M2 macrophages exert a therapeutic effect via inhibition of the PI3K/AKT/mTOR pathway in rats with knee osteoarthritic. Biomed Res Int. 2021;2021:7218067. 34926690 10.1155/2021/7218067 PMC8683166

[53] Li P , Lv S , Jiang W , Si L , Liao B , Zhao G , et al. Exosomes derived from umbilical cord mesenchymal stem cells protect cartilage and regulate the polarization of macrophages in osteoarthritis. Ann Transl Med. 2022;10(18):976. 36267713 10.21037/atm-22-3912 PMC9577719

[54] Wu J , Kuang L , Chen C , Yang J , Zeng WN , Li T , et al. miR‐100‐5p‐abundant exosomes derived from infrapatellar fat pad MSCs protect articular cartilage and ameliorate gait abnormalities via inhibition of mTOR in osteoarthritis. Biomaterials. 2019;206:87–100. 30927715 10.1016/j.biomaterials.2019.03.022

[55] Xu J , Ye Z , Han K , Zheng T , Zhang T , Dong S , et al. Infrapatellar fat pad mesenchymal stromal cell‐derived exosomes accelerate tendon‐bone healing and intra‐articular graft remodeling after anterior cruciate ligament reconstruction. Am J Sports Med. 2022;50(3):662–673. 35224997 10.1177/03635465211072227

[56] Chen Y , Wang T , Guan M , Zhao W , Leung FKL , Pan H , et al. Bone turnover and articular cartilage differences localized to subchondral cysts in knees with advanced osteoarthritis. Osteoarthr Cartil. 2015;23(12):2174–2183. 10.1016/j.joca.2015.07.012 26241776

[57] Day JS , Ding M , van der Linden JC , Hvid I , Sumner DR , Weinans H . A decreased subchondral trabecular bone tissue elastic modulus is associated with pre‐arthritic cartilage damage. J Orthop Res. 2001;19(5):914–918. 11562141 10.1016/S0736-0266(01)00012-2

[58] Prieto‐Potin I , Largo R , Roman‐Blas JA , Herrero‐Beaumont G , Walsh DA . Characterization of multinucleated giant cells in synovium and subchondral bone in knee osteoarthritis and rheumatoid arthritis. BMC Musculoskelet Disord. 2015;16:226. 26311062 10.1186/s12891-015-0664-5 PMC4550054

[59] Geurts J , Patel A , Hirschmann MT , Pagenstert GI , Müller‐Gerbl M , Valderrabano V , et al. Elevated marrow inflammatory cells and osteoclasts in subchondral osteosclerosis in human knee osteoarthritis. J Orthop Res. 2016;34(2):262–269. 26250062 10.1002/jor.23009

[60] Li G , Yin J , Gao J , Cheng TS , Pavlos NJ , Zhang C , et al. Subchondral bone in osteoarthritis: insight into risk factors and microstructural changes. Arthritis Res Ther. 2013;15(6):223. 24321104 10.1186/ar4405 PMC4061721

[61] Su W , Liu G , Liu X , Zhou Y , Sun Q , Zhen G , et al. Angiogenesis stimulated by elevated PDGF‐BB in subchondral bone contributes to osteoarthritis development. JCI Insight. 2020;5(8):e135446. 32208385 10.1172/jci.insight.135446 PMC7205438

[62] Jiang G , Li S , Yu K , He B , Hong J , Xu T , et al. A 3D‐printed PRP‐GelMA hydrogel promotes osteochondral regeneration through M2 macrophage polarization in a rabbit model. Acta Biomater. 2021;128:150–162. 33894346 10.1016/j.actbio.2021.04.010

[63] Taniguchi K , Karin M . NF‐kappaB, inflammation, immunity and cancer: coming of age. Nat Rev Immunol. 2018;18(5):309–324. 29379212 10.1038/nri.2017.142

[64] Zhang Q , Lenardo MJ , Baltimore D . 30 years of NF‐kappaB: a blossoming of relevance to human pathobiology. Cell. 2017;168(1–2):37–57. 28086098 10.1016/j.cell.2016.12.012 PMC5268070

[65] Sun SC . The non‐canonical NF‐kappaB pathway in immunity and inflammation. Nat Rev Immunol. 2017;17(9):545–558. 28580957 10.1038/nri.2017.52 PMC5753586

[66] Capece D , Verzella D , Flati I , Arboretto P , Cornice J , Franzoso G . NF‐kappaB: blending metabolism, immunity, and inflammation. Trends Immunol. 2022;43(9):757–775. 35965153 10.1016/j.it.2022.07.004

[67] Napetschnig J , Wu H . Molecular basis of NF‐kappaB signaling. Annu Rev Biophys. 2013;42:443–468. 23495970 10.1146/annurev-biophys-083012-130338 PMC3678348

[68] Hayden MS , Ghosh S . Shared principles in NF‐kappaB signaling. Cell. 2008;132(3):344–362. 18267068 10.1016/j.cell.2008.01.020

[69] Yu H , Lin L , Zhang Z , Zhang H , Hu H . Targeting NF‐kappaB pathway for the therapy of diseases: mechanism and clinical study. Signal Transduct Target Ther. 2020;5(1):209. 32958760 10.1038/s41392-020-00312-6 PMC7506548

[70] Lee CH , Chiang CF , Kuo FC , Su SC , Huang CL , Liu JS , et al. High‐molecular‐weight hyaluronic acid inhibits IL‐1β‐induced synovial inflammation and macrophage polarization through the GRP78‐NF‐κB signaling pathway. Int J Mol Sci. 2021;22(21):12035. 34769349 10.3390/ijms222111917 PMC8584972

[71] Lu Y , Liu L , Pan J , Luo B , Zeng H , Shao Y , et al. MFG‐E8 regulated by miR‐99b‐5p protects against osteoarthritis by targeting chondrocyte senescence and macrophage reprogramming via the NF‐kappaB pathway. Cell Death Dis. 2021;12(6):533. 34031369 10.1038/s41419-021-03800-x PMC8144578

[72] Jiang H , Zhang Y , Hu G , Shang X , Ming J , Deng M , et al. Innate/inflammatory bioregulation of surfactant protein D alleviates rat osteoarthritis by inhibiting toll‐like receptor 4 signaling. Front Immunol. 2022;13:913901. 35865531 10.3389/fimmu.2022.913901 PMC9294227

[73] Gong J , Li J , Dong H , Chen G , Qin X , Hu M , et al. Inhibitory effects of berberine on proinflammatory M1 macrophage polarization through interfering with the interaction between TLR4 and MyD88. BMC Complement Altern Med. 2019;19(1):314. 31744490 10.1186/s12906-019-2710-6 PMC6862859

[74] Mo H , Wang Z , He Z , Wan J , Lu R , Wang C , et al. Decreased Peli1 expression attenuates osteoarthritis by protecting chondrocytes and inhibiting M1‐polarization of macrophages. Bone Joint Res. 2023;12(2):121–132. 36718653 10.1302/2046-3758.122.BJR-2022-0214.R1 PMC9950670

[75] Barreto G , Senturk B , Colombo L , Brück O , Neidenbach P , Salzmann G , et al. Lumican is upregulated in osteoarthritis and contributes to TLR4‐induced pro‐inflammatory activation of cartilage degradation and macrophage polarization. Osteoarthr Cartil. 2020;28(1):92–101. 10.1016/j.joca.2019.10.011 31715293

[76] Musumeci G , Castrogiovanni P , Trovato F , Weinberg A , al‐Wasiyah M , Alqahtani M , et al. Biomarkers of chondrocyte apoptosis and autophagy in osteoarthritis. Int J Mol Sci. 2015;16(9):20560–20575. 26334269 10.3390/ijms160920560 PMC4613218

[77] Li J , Ye F , Xu X , Xu P , Wang P , Zheng G , et al. Targeting macrophage M1 polarization suppression through PCAF inhibition alleviates autoimmune arthritis via synergistic NF‐κB and H3K9Ac blockade. J Nanobiotechnol. 2023;21(1):280. 10.1186/s12951-023-02012-z PMC10439630 37598147

[78] Yunna C , Mengru H , Lei W , Weidong C . Macrophage M1/M2 polarization. Eur J Pharmacol. 2020;877:173090. 32234529 10.1016/j.ejphar.2020.173090

[79] Chung SW , Kang BY , Kim SH , Pak YK , Cho D , Trinchieri G , et al. Oxidized low density lipoprotein inhibits interleukin‐12 production in lipopolysaccharide‐activated mouse macrophages via direct interactions between peroxisome proliferator‐activated receptor‐gamma and nuclear factor‐kappa B. J Biol Chem. 2000;275(42):32681–32687. 10934192 10.1074/jbc.M002577200

[80] Hata A , Chen YG . TGF‐beta signaling from receptors to Smads. Cold Spring Harb Perspect Biol. 2016;8(9):a022061. 27449815 10.1101/cshperspect.a022061 PMC5008074

[81] Derynck R , Budi EH . Specificity, versatility, and control of TGF‐beta family signaling. Sci Signal. 2019;12(570):eaav5183. 30808818 10.1126/scisignal.aav5183 PMC6800142

[82] Chen G , Deng C , Li YP . TGF‐beta and BMP signaling in osteoblast differentiation and bone formation. Int J Biol Sci. 2012;8(2):272–288. 22298955 10.7150/ijbs.2929 PMC3269610

[83] Lamouille S , Xu J , Derynck R . Molecular mechanisms of epithelial‐mesenchymal transition. Nat Rev Mol Cell Biol. 2014;15(3):178–196. 24556840 10.1038/nrm3758 PMC4240281

[84] Yi JJ , Barnes AP , Hand R , Polleux F , Ehlers MD . TGF‐beta signaling specifies axons during brain development. Cell. 2010;142(1):144–157. 20603020 10.1016/j.cell.2010.06.010 PMC2933408

[85] Perez LG , Kempski J , McGee HM , Pelzcar P , Agalioti T , Giannou A , et al. TGF‐beta signaling in Th17 cells promotes IL‐22 production and colitis‐associated colon cancer. Nat Commun. 2020;11(1):2608. 32451418 10.1038/s41467-020-16363-w PMC7248087

[86] Itoh S , ten Dijke P . Negative regulation of TGF‐beta receptor/Smad signal transduction. Curr Opin Cell Biol. 2007;19(2):176–184. 17317136 10.1016/j.ceb.2007.02.015

[87] Li D , Zhang Q , Li L , Chen K , Yang J , Dixit D , et al. β2‐microglobulin maintains glioblastoma stem cells and induces M2‐like polarization of tumor‐associated macrophages. Cancer Res. 2022;82(18):3321–3334. 35841593 10.1158/0008-5472.CAN-22-0507

[88] Zhu X , Liang R , Lan T , Ding D , Huang S , Shao J , et al. Tumor‐associated macrophage‐specific CD155 contributes to M2‐phenotype transition, immunosuppression, and tumor progression in colorectal cancer. J Immuno Ther Cancer. 2022;10(9):e004219. 10.1136/jitc-2021-004219 PMC9476138 36104099

[89] Ma C , He D , Tian P , Wang Y , He Y , Wu Q , et al. miR‐182 targeting reprograms tumor‐associated macrophages and limits breast cancer progression. Proc Natl Acad Sci. 2022;119(6):e2114006119. 35105806 10.1073/pnas.2114006119 PMC8833194

[90] Dai M , Sui B , Xue Y , Liu X , Sun J . Cartilage repair in degenerative osteoarthritis mediated by squid type II collagen via immunomodulating activation of M2 macrophages, inhibiting apoptosis and hypertrophy of chondrocytes. Biomaterials. 2018;180:91–103. 30031224 10.1016/j.biomaterials.2018.07.011

[91] Sun Y , Huang K , Mo L , Ahmad A , Wang D , Rong Z , et al. Eucommia ulmoides polysaccharides attenuate rabbit osteoarthritis by regulating the function of macrophages. Front Pharmacol. 2021;12:730557. 34421623 10.3389/fphar.2021.730557 PMC8377595

[92] Lee H , Kim H , Seo J , Choi K , Lee Y , Park K , et al. TissueGene‐C promotes an anti‐inflammatory micro‐environment in a rat monoiodoacetate model of osteoarthritis via polarization of M2 macrophages leading to pain relief and structural improvement. Inflammopharmacology. 2020;28(5):1237–1252. 32696209 10.1007/s10787-020-00738-y PMC7524813

[93] Wang Q , Feng J , Tang L . Non‐coding RNA related to MAPK signaling pathway in liver cancer. Int J Mol Sci. 2022;23(19):11908. 10.3390/ijms231911908 PMC9570382 36233210

[94] Fang JY , Richardson BC . The MAPK signalling pathways and colorectal cancer. Lancet Oncol. 2005;6(5):322–327. 15863380 10.1016/S1470-2045(05)70168-6

[95] Lee S , Rauch J , Kolch W . Targeting MAPK signaling in cancer: mechanisms of drug resistance and sensitivity. Int J Mol Sci. 2020;21(3):1102. 10.3390/ijms21031102 PMC7037308 32046099

[96] Asl ER , Amini M , Najafi S , Mansoori B , Mokhtarzadeh A , Mohammadi A , et al. Interplay between MAPK/ERK signaling pathway and MicroRNAs: a crucial mechanism regulating cancer cell metabolism and tumor progression. Life Sci. 2021;278:119499. 33865878 10.1016/j.lfs.2021.119499

[97] Hepworth EMW , Hinton SD . Pseudophosphatases as regulators of MAPK signaling. Int J Mol Sci. 2021;22(22):12595. 34830476 10.3390/ijms222212595 PMC8622459

[98] Zheng X , Jiang Q , Han M , Ye F , Wang M , Qiu Y , et al. FBXO38 regulates macrophage polarization to control the development of cancer and colitis. Cell Mol Immunol. 2023;20(11):1367–1378. 37821621 10.1038/s41423-023-01081-2 PMC10616184

[99] Qiu S , Xie L , Lu C , Gu C , Xia Y , Lv J , et al. Gastric cancer‐derived exosomal miR‐519a‐3p promotes liver metastasis by inducing intrahepatic M2‐like macrophage‐mediated angiogenesis. J Exp Clin Cancer Res. 2022;41(1):296. 36217165 10.1186/s13046-022-02499-8 PMC9549645

[100] Moon SM , Lee SA , Han SH , Park BR , Choi MS , Kim JS , et al. Aqueous extract of Codium fragile alleviates osteoarthritis through the MAPK/NF‐κB pathways in IL‐1β‐induced rat primary chondrocytes and a rat osteoarthritis model. Biomed Pharmacother. 2018;97:264–270. 29091874 10.1016/j.biopha.2017.10.130

[101] Ran J , Ma C , Xu K , Xu L , He Y , Moqbel SAA , et al. Schisandrin B ameliorated chondrocytes inflammation and osteoarthritis via suppression of NF‐κB and MAPK signal pathways. Drug Des Devel Ther. 2018;12:1195–1204. 10.2147/DDDT.S162014 PMC5953308 29785089

[102] Wu MH , Tsai CH , Huang YL , Fong YC , Tang CH . Visfatin promotes IL‐6 and TNF‐α production in human synovial fibroblasts by repressing miR‐199a‐5p through ERK, p38 and JNK signaling pathways. Int J Mol Sci. 2018;19(1):190. 10.3390/ijms19010190 PMC5796139 29316707

[103] Zhou F , Mei J , Han X , Li H , Yang S , Wang M , et al. Kinsenoside attenuates osteoarthritis by repolarizing macrophages through inactivating NF‐κB/MAPK signaling and protecting chondrocytes. Acta Pharm Sin B. 2019;9(5):973–985. 31649847 10.1016/j.apsb.2019.01.015 PMC6804452

[104] Mahon OR , O'Hanlon S , Cunningham CC , McCarthy GM , Hobbs C , Nicolosi V , et al. Orthopaedic implant materials drive M1 macrophage polarization in a spleen tyrosine kinase‐ and mitogen‐activated protein kinase‐dependent manner. Acta Biomater. 2018;65:426–435. 29104084 10.1016/j.actbio.2017.10.041

[105] Philips RL , Wang Y , Cheon HJ , Kanno Y , Gadina M , Sartorelli V , et al. The JAK‐STAT pathway at 30: much learned, much more to do. Cell. 2022;185(21):3857–3876. 36240739 10.1016/j.cell.2022.09.023 PMC9815833

[106] Tanaka Y , Luo Y , O'Shea JJ , Nakayamada S . Janus kinase‐targeting therapies in rheumatology: a mechanisms‐based approach. Nat Rev Rheumatol. 2022;18(3):133–145. 34987201 10.1038/s41584-021-00726-8 PMC8730299

[107] Hu X , li J , Fu M , Zhao X , Wang W . The JAK/STAT signaling pathway: from bench to clinic. Signal Transduct Target Ther. 2021;6(1):402. 34824210 10.1038/s41392-021-00791-1 PMC8617206

[108] Garcia S , Krausz S , Ambarus CA , Fernández BM , Hartkamp LM , van Es IE , et al. Tie2 signaling cooperates with TNF to promote the pro‐inflammatory activation of human macrophages independently of macrophage functional phenotype. PloS One. 2014;9(1):e82088. 24404127 10.1371/journal.pone.0082088 PMC3880273

[109] Wang Q , Zhou X , Yang L , Zhao Y , Chew Z , Xiao J , et al. The natural compound Notopterol binds and targets JAK2/3 to ameliorate inflammation and arthritis. Cell Rep. 2020;32(11):108158. 32937124 10.1016/j.celrep.2020.108158

[110] De Vries LCS , Duarte JM , De Krijger M , Welting O , Van Hamersveld PH , Van Leeuwen‐Hilbers FW , et al. A JAK1 selective kinase inhibitor and tofacitinib affect macrophage activation and function. Inflamm Bowel Dis. 2019;25(4):647–660. 30668755 10.1093/ibd/izy364

[111] Xu J , Zhang J , Zhang Z , Gao Z , Qi Y , Qiu W , et al. Hypoxic glioma‐derived exosomes promote M2‐like macrophage polarization by enhancing autophagy induction. Cell Death Dis. 2021;12(4):373. 33828078 10.1038/s41419-021-03664-1 PMC8026615

[112] Xu M , Li X , Song L . Baicalin regulates macrophages polarization and alleviates myocardial ischaemia/reperfusion injury via inhibiting JAK/STAT pathway. Pharm Biol. 2020;58(1):655–663. 32649845 10.1080/13880209.2020.1779318 PMC7470075

[113] Liu Y , Wang L , Li S , Zhang T , Chen C , Hu J , et al. Mechanical stimulation improves rotator cuff tendon‐bone healing via activating IL‐4/JAK/STAT signaling pathway mediated macrophage M2 polarization. J Orthop Translat. 2022;37:78–88. 36262964 10.1016/j.jot.2022.08.008 PMC9550856

[114] Degboe Y , Rauwel B , Baron M , Boyer JF , Ruyssen‐Witrand A , Constantin A , et al. Polarization of rheumatoid macrophages by TNF targeting through an IL‐10/STAT3 mechanism. Front Immunol. 2019;10:3. 30713533 10.3389/fimmu.2019.00003 PMC6345709

[115] Zhang MZ , Wang X , Wang Y , Niu A , Wang S , Zou C , et al. IL‐4/IL‐13‐mediated polarization of renal macrophages/dendritic cells to an M2a phenotype is essential for recovery from acute kidney injury. Kidney Int. 2017;91(2):375–386. 27745702 10.1016/j.kint.2016.08.020 PMC5548101

[116] Bhattacharjee A , Shukla M , Yakubenko VP , Mulya A , Kundu S , Cathcart MK . IL‐4 and IL‐13 employ discrete signaling pathways for target gene expression in alternatively activated monocytes/macrophages. Free Radic Biol Med. 2013;54:1–16. 23124025 10.1016/j.freeradbiomed.2012.10.553 PMC3534796

[117] Durham GA , Williams JJL , Nasim MT , Palmer TM . Targeting SOCS proteins to control JAK‐STAT Signalling in disease. Trends Pharmacol Sci. 2019;40(5):298–308. 30948191 10.1016/j.tips.2019.03.001

[118] Liang YB , Tang H , Chen ZB , Zeng LJ , Wu JG , Yang W , et al. Downregulated SOCS1 expression activates the JAK1/STAT1 pathway and promotes polarization of macrophages into M1 type. Mol Med Rep. 2017;16(5):6405–6411. 28901399 10.3892/mmr.2017.7384

[119] Wang F , Zhang S , Vuckovic I , Jeon R , Lerman A , Folmes CD , et al. Glycolytic stimulation is not a requirement for M2 macrophage differentiation. Cell Metab. 2018;28(3):463–475.e4. 30184486 10.1016/j.cmet.2018.08.012 PMC6449248

[120] Ji L , Zhao X , Zhang B , Kang L , Song W , Zhao B , et al. Slc6a8‐mediated creatine uptake and accumulation reprogram macrophage polarization via regulating cytokine responses. Immunity. 2019;51(2):272–284.e7. 31399282 10.1016/j.immuni.2019.06.007

[121] Shan X , Hu P , Ni L , Shen L , Zhang Y , Ji Z , et al. Serine metabolism orchestrates macrophage polarization by regulating the IGF1‐p38 axis. Cell Mol Immunol. 2022;19(11):1263–1278. 36180780 10.1038/s41423-022-00925-7 PMC9622887

[122] Latourte A , Cherifi C , Maillet J , Ea HK , Bouaziz W , Funck‐Brentano T , et al. Systemic inhibition of IL‐6/Stat3 signalling protects against experimental osteoarthritis. Ann Rheum Dis. 2017;76(4):748–755. 27789465 10.1136/annrheumdis-2016-209757

[123] Zhou Q , Ren Q , Jiao L , Huang J , Yi J , Chen J , et al. The potential roles of JAK/STAT signaling in the progression of osteoarthritis. Front Endocrinol (Lausanne). 2022;13:1069057. 36506076 10.3389/fendo.2022.1069057 PMC9729341

[124] Hu Y , Gui Z , Zhou Y , Xia L , Lin K , Xu Y . Quercetin alleviates rat osteoarthritis by inhibiting inflammation and apoptosis of chondrocytes, modulating synovial macrophages polarization to M2 macrophages. Free Radic Biol Med. 2019;145:146–160. 31550528 10.1016/j.freeradbiomed.2019.09.024

[125] Di‐Luoffo M , Ben‐Meriem Z , Lefebvre P , Delarue M , Guillermet‐Guibert J . PI3K functions as a hub in mechanotransduction. Trends Biochem Sci. 2021;46(11):878–888. 34112586 10.1016/j.tibs.2021.05.005

[126] Fruman DA , Chiu H , Hopkins BD , Bagrodia S , Cantley LC , Abraham RT . The PI3K pathway in human disease. Cell. 2017;170(4):605–635. 28802037 10.1016/j.cell.2017.07.029 PMC5726441

[127] Yang J , Nie J , Ma X , Wei Y , Peng Y , Wei X . Targeting PI3K in cancer: mechanisms and advances in clinical trials. Mol Cancer. 2019;18(1):26. 30782187 10.1186/s12943-019-0954-x PMC6379961

[128] Geraldo LH , Xu Y , Jacob L , Pibouin‐Fragner L , Rao R , Maissa N , et al. SLIT2/ROBO signaling in tumor‐associated microglia and macrophages drives glioblastoma immunosuppression and vascular dysmorphia. J Clin Invest. 2021;131(16):e141083. 10.1172/JCI141083 PMC8363292 34181595

[129] Wang Y , Lyu Z , Qin Y , Wang X , Sun L , Zhang Y , et al. FOXO1 promotes tumor progression by increased M2 macrophage infiltration in esophageal squamous cell carcinoma. Theranostics. 2020;10(25):11535–11548. 33052231 10.7150/thno.45261 PMC7546008

[130] Fu W , Hu W , Yi YS , Hettinghouse A , Sun G , Bi Y , et al. TNFR2/14‐3‐3ε signaling complex instructs macrophage plasticity in inflammation and autoimmunity. J Clin Invest. 2021;131(16):e144016. 10.1172/JCI144016 PMC8363273 34185706

[131] Wu J , Zhang L , Shi J , He R , Yang W , Habtezion A , et al. Macrophage phenotypic switch orchestrates the inflammation and repair/regeneration following acute pancreatitis injury. EBioMedicine. 2020;58:102920. 32739869 10.1016/j.ebiom.2020.102920 PMC7399125

[132] Zhang H , Cai D , Bai X . Macrophages regulate the progression of osteoarthritis. Osteoarthr Cartil. 2020;28(5):555–561. 10.1016/j.joca.2020.01.007 31982565

[133] Sun K , Luo J , Guo J , Yao X , Jing X , Guo F . The PI3K/AKT/mTOR signaling pathway in osteoarthritis: a narrative review. Osteoarthr Cartil. 2020;28(4):400–409. 10.1016/j.joca.2020.02.027 32081707

[134] Liu X , Chen M , Luo J , Zhao H , Zhou X , Gu Q , et al. Immunopolarization‐regulated 3D printed‐electrospun fibrous scaffolds for bone regeneration. Biomaterials. 2021;276:121037. 34325336 10.1016/j.biomaterials.2021.121037

[135] Li K , Yan G , Huang H , Zheng M , Ma K , Cui X , et al. Anti‐inflammatory and immunomodulatory effects of the extracellular vesicles derived from human umbilical cord mesenchymal stem cells on osteoarthritis via M2 macrophages. J Nanobiotechnology. 2022;20(1):38. 35057811 10.1186/s12951-021-01236-1 PMC8771624

[136] Zheng M , Zhu Y , Wei K , Pu H , Peng R , Xiao J , et al. Metformin attenuates the inflammatory response via the regulation of synovial M1 macrophage in osteoarthritis. Int J Mol Sci. 2023;24(6):5206. 36982442 10.3390/ijms24065355 PMC10049635

[137] Wang L , Hauenstein AV . The NLRP3 inflammasome: mechanism of action, role in disease and therapies. Mol Aspects Med. 2020;76:100889. 32859386 10.1016/j.mam.2020.100889

[138] Mangan MSJ , Olhava EJ , Roush WR , Seidel HM , Glick GD , Latz E . Targeting the NLRP3 inflammasome in inflammatory diseases. Nat Rev Drug Discov. 2018;17(8):588–606. 30026524 10.1038/nrd.2018.97

[139] Akbal A , Dernst A , Lovotti M , Mangan MSJ , McManus RM , Latz E . How location and cellular signaling combine to activate the NLRP3 inflammasome. Cell Mol Immunol. 2022;19(11):1201–1214. 36127465 10.1038/s41423-022-00922-w PMC9622870

[140] Paik S , Kim JK , Silwal P , Sasakawa C , Jo EK . An update on the regulatory mechanisms of NLRP3 inflammasome activation. Cell Mol Immunol. 2021;18(5):1141–1160. 33850310 10.1038/s41423-021-00670-3 PMC8093260

[141] Swanson KV , Deng M , Ting JP . The NLRP3 inflammasome: molecular activation and regulation to therapeutics. Nat Rev Immunol. 2019;19(8):477–489. 31036962 10.1038/s41577-019-0165-0 PMC7807242

[142] Huang Y , Xu W , Zhou R . NLRP3 inflammasome activation and cell death. Cell Mol Immunol. 2021;18(9):2114–2127. 34321623 10.1038/s41423-021-00740-6 PMC8429580

[143] McAllister MJ , Chemaly M , Eakin AJ , Gibson DS , McGilligan VE . NLRP3 as a potentially novel biomarker for the management of osteoarthritis. Osteoarthr Cartil. 2018;26(5):612–619. 10.1016/j.joca.2018.02.901 29499288

[144] Wisitpongpun P , Potup P , Usuwanthim K . Oleamide‐mediated polarization of M1 macrophages and IL‐1beta production by regulating NLRP3‐inflammasome activation in primary human monocyte‐derived macrophages. Front Immunol. 2022;13:856296. 35514993 10.3389/fimmu.2022.856296 PMC9062104

[145] Liu T , Wang L , Liang P , Wang X , Liu Y , Cai J , et al. USP19 suppresses inflammation and promotes M2‐like macrophage polarization by manipulating NLRP3 function via autophagy. Cell Mol Immunol. 2021;18(10):2431–2442. 33097834 10.1038/s41423-020-00567-7 PMC8484569

[146] Sun H , Sun Z , Xu X , Lv Z , Li J , Wu R , et al. Blocking TRPV4 ameliorates osteoarthritis by inhibiting M1 macrophage polarization via the ROS/NLRP3 signaling pathway. Antioxidants (Basel). 2022;11(12):2315. 10.3390/antiox11122315 PMC9774183 36552524

[147] Luo P , Peng S , Yan Y , Ji P , Xu J . IL‐37 inhibits M1‐like macrophage activation to ameliorate temporomandibular joint inflammation through the NLRP3 pathway. Rheumatology (Oxford). 2020;59(10):3070–3080. 32417913 10.1093/rheumatology/keaa192

[148] Shen P , Jia S , Wang Y , Zhou X , Zhang D , Jin Z , et al. Mechanical stress protects against chondrocyte pyroptosis through lipoxin a(4) via synovial macrophage M2 subtype polarization in an osteoarthritis model. Biomed Pharmacother. 2022;153:113361. 35797941 10.1016/j.biopha.2022.113361

[149] Sun Y , Zuo Z , Kuang Y . An emerging target in the Battle against osteoarthritis: macrophage polarization. Int J Mol Sci. 2020;21(22):8513. 33198196 10.3390/ijms21228513 PMC7697192

[150] Ni L , Lin Z , Hu S , Shi Y , Jiang Z , Zhao J , et al. Itaconate attenuates osteoarthritis by inhibiting STING/NF‐kappaB axis in chondrocytes and promoting M2 polarization in macrophages. Biochem Pharmacol. 2022;198:114935. 35104478 10.1016/j.bcp.2022.114935

[151] Khatab S , van Buul GM , Kops N , Bastiaansen‐Jenniskens YM , Bos PK , Verhaar JA , et al. Intra‐articular injections of platelet‐Rich plasma Releasate reduce pain and synovial inflammation in a mouse model of osteoarthritis. Am J Sports Med. 2018;46(4):977–986. 29373806 10.1177/0363546517750635

[152] Lu J , Zhang H , Pan J , Hu Z , Liu L , Liu Y , et al. Fargesin ameliorates osteoarthritis via macrophage reprogramming by downregulating MAPK and NF‐kappaB pathways. Arthritis Res Ther. 2021;23(1):142. 33990219 10.1186/s13075-021-02512-z PMC8120707

[153] Wang H , Zhang H , Fan K , Zhang D , Hu A , Zeng X , et al. Frugoside delays osteoarthritis progression via inhibiting miR‐155‐modulated synovial macrophage M1 polarization. Rheumatology (Oxford). 2021;60(10):4899–4909. 33493345 10.1093/rheumatology/keab018

[154] Zhang J , Rong Y , Luo C , Cui W . Bone marrow mesenchymal stem cell‐derived exosomes prevent osteoarthritis by regulating synovial macrophage polarization. Aging (Albany NY). 2020;12(24):25138–25152. 33350983 10.18632/aging.104110 PMC7803581

[155] Manferdini C , Paolella F , Gabusi E , Gambari L , Piacentini A , Filardo G , et al. Adipose stromal cells mediated switching of the pro‐inflammatory profile of M1‐like macrophages is facilitated by PGE2: in vitro evaluation. Osteoarthr Cartil. 2017;25(7):1161–1171. 10.1016/j.joca.2017.01.011 28153787

[156] Ma Y , Yang H , Zong X , Wu J , Ji X , Liu W , et al. Artificial M2 macrophages for disease‐modifying osteoarthritis therapeutics. Biomaterials. 2021;274:120865. 33991950 10.1016/j.biomaterials.2021.120865

[157] Zhang L , Chen X , Cai P , Sun H , Shen S , Guo B , et al. Reprogramming mitochondrial metabolism in synovial macrophages of early osteoarthritis by a camouflaged meta‐Defensome. Adv Mater. 2022;34(30):e2202715. 35671349 10.1002/adma.202202715

[158] Zhou F , Mei J , Yang S , Han X , Li H , Yu Z , et al. Modified ZIF‐8 nanoparticles attenuate osteoarthritis by reprogramming the metabolic pathway of synovial macrophages. ACS Appl Mater Interfaces. 2020;12(2):2009–2022. 31849213 10.1021/acsami.9b16327

[159] Castorina S , Guglielmino C , Castrogiovanni P , Szychlinska MA , Ioppolo F , Massimino P , et al. Clinical evidence of traditional vs fast track recovery methodologies after total arthroplasty for osteoarthritic knee treatment. A retrospective observational study. Muscles Ligaments Tendons J. 2017;7(3):504–513. 29387645 10.11138/mltj/2017.7.3.504 PMC5774925

[160] Bull FC , al‐Ansari SS , Biddle S , Borodulin K , Buman MP , Cardon G , et al. World Health Organization 2020 guidelines on physical activity and sedentary behaviour. Br J Sports Med. 2020;54(24):1451–1462. 33239350 10.1136/bjsports-2020-102955 PMC7719906

[161] Gill SD , McBurney H . Does exercise reduce pain and improve physical function before hip or knee replacement surgery? A systematic review and meta‐analysis of randomized controlled trials. Arch Phys Med Rehabil. 2013;94(1):164–176. 22960276 10.1016/j.apmr.2012.08.211

[162] Shan L , Shan B , Suzuki A , Nouh F , Saxena A . Intermediate and long‐term quality of life after total knee replacement: a systematic review and meta‐analysis. J Bone Joint Surg Am. 2015;97(2):156–168. 25609443 10.2106/JBJS.M.00372

[163] Silveira LS , Batatinha HAP , Castoldi A , Câmara NOS , Festuccia WT , Souza CO , et al. Exercise rescues the immune response fine‐tuned impaired by peroxisome proliferator‐activated receptors gamma deletion in macrophages. J Cell Physiol. 2019;234(4):5241–5251. 30238979 10.1002/jcp.27333

[164] Abbasi J . Can exercise prevent knee osteoarthritis? Jama. 2017;318(22):2169–2171. 29167894 10.1001/jama.2017.16144

[165] Emery CA , Pasanen K . Current trends in sport injury prevention. Best Pract Res Clin Rheumatol. 2019;33(1):3–15. 31431273 10.1016/j.berh.2019.02.009

[166] Liao B , Guan M , Tan Q , Wang G , Zhang R , Huang J , et al. Low‐intensity pulsed ultrasound inhibits fibroblast‐like synoviocyte proliferation and reduces synovial fibrosis by regulating Wnt/β‐catenin signaling. J Orthop Translat. 2021;30:41–50. 34611513 10.1016/j.jot.2021.08.002 PMC8458725

[167] Uddin SMZ , Komatsu DE . Therapeutic potential Low‐intensity pulsed ultrasound for osteoarthritis: pre‐clinical and clinical perspectives. Ultrasound Med Biol. 2020;46(4):909–920. 31959508 10.1016/j.ultrasmedbio.2019.12.007

[168] Xu Z , Li S , Wan L , Hu J , Lu H , Zhang T . Role of low‐intensity pulsed ultrasound in regulating macrophage polarization to accelerate tendon‐bone interface repair. J Orthop Res. 2022;41:919–929. 36203341 10.1002/jor.25454

[169] Letizia Mauro G , Scaturro D , Gimigliano F , Paoletta M , Liguori S , Toro G , et al. Physical agent modalities in early osteoarthritis: a scoping review. Medicina (Kaunas). 2021;57(11):1165. 10.3390/medicina57111165 PMC8619194 34833383

[170] Arden NK , Perry TA , Bannuru RR , Bruyère O , Cooper C , Haugen IK , et al. Non‐surgical management of knee osteoarthritis: comparison of ESCEO and OARSI 2019 guidelines. Nat Rev Rheumatol. 2021;17(1):59–66. 33116279 10.1038/s41584-020-00523-9

[171] Shu CC , Zaki S , Ravi V , Schiavinato A , Smith MM , Little CB . The relationship between synovial inflammation, structural pathology, and pain in post‐traumatic osteoarthritis: differential effect of stem cell and hyaluronan treatment. Arthritis Res Ther. 2020;22(1):29. 32059749 10.1186/s13075-020-2117-2 PMC7023816

[172] Tarricone E , Mattiuzzo E , Belluzzi E , Elia R , Benetti A , Venerando R , et al. Anti‐inflammatory performance of lactose‐modified chitosan and hyaluronic acid mixtures in an in vitro macrophage‐mediated inflammation osteoarthritis model. Cell. 2020;9(6):1328. 10.3390/cells9061328 PMC7349682 32466461

[173] Jin L , Xu K , Liang Y , du P , Wan S , Jiang C . Effect of hyaluronic acid on cytokines and immune cells change in patients of knee osteoarthritis. BMC Musculoskelet Disord. 2022;23(1):812. 36008806 10.1186/s12891-022-05767-y PMC9404574

[174] Rayahin JE , Buhrman JS , Zhang Y , Koh TJ , Gemeinhart RA . High and low molecular weight hyaluronic acid differentially influence macrophage activation. ACS Biomater Sci Eng. 2015;1(7):481–493. 26280020 10.1021/acsbiomaterials.5b00181 PMC4533115

[175] Uçar D , Dıraçoğlu D , Süleyman T , Çapan N . Intra‐articular hyaluronic acid as treatment in elderly and middle‐aged patients with knee osteoarthritis. Open Rheumatol J. 2013;7:38–41. 23919093 10.2174/1874312901307010038 PMC3731797

[176] Lohmander LS , Dalen N , Englund G , Hamalainen M , Jensen EM , Karlsson K , et al. Intra‐articular hyaluronan injections in the treatment of osteoarthritis of the knee: a randomised, double blind, placebo controlled multicentre trial. Hyaluronan multicentre trial group. Ann Rheum Dis. 1996;55(7):424–431. 8774159 10.1136/ard.55.7.424 PMC1010204

[177] Perl A . Activation of mTOR (mechanistic target of rapamycin) in rheumatic diseases. Nat Rev Rheumatol. 2016;12(3):169–182. 26698023 10.1038/nrrheum.2015.172 PMC5314913

[178] Shan M , Qin J , Jin F , Han X , Guan H , Li X , et al. Autophagy suppresses isoprenaline‐induced M2 macrophage polarization via the ROS/ERK and mTOR signaling pathway. Free Radic Biol Med. 2017;110:432–443. 28647611 10.1016/j.freeradbiomed.2017.05.021

[179] Vergadi E , Ieronymaki E , Lyroni K , Vaporidi K , Tsatsanis C . Akt signaling pathway in macrophage activation and M1/M2 polarization. J Immunol. 2017;198(3):1006–1014. 28115590 10.4049/jimmunol.1601515

[180] Kang S , Nakanishi Y , Kioi Y , Okuzaki D , Kimura T , Takamatsu H , et al. Semaphorin 6D reverse signaling controls macrophage lipid metabolism and anti‐inflammatory polarization. Nat Immunol. 2018;19(6):561–570. 29777213 10.1038/s41590-018-0108-0

[181] Wu MM , Wang QM , Huang BY , Mai CT , Wang CL , Wang TT , et al. Dioscin ameliorates murine ulcerative colitis by regulating macrophage polarization. Pharmacol Res. 2021;172:105796. 34343656 10.1016/j.phrs.2021.105796

[182] Xu X , Gao W , Li L , Hao J , Yang B , Wang T , et al. Annexin A1 protects against cerebral ischemia‐reperfusion injury by modulating microglia/macrophage polarization via FPR2/ALX‐dependent AMPK‐mTOR pathway. J Neuroinflammation. 2021;18(1):119. 34022892 10.1186/s12974-021-02174-3 PMC8140477

[183] Hooftman A , Angiari S , Hester S , Corcoran SE , Runtsch MC , Ling C , et al. The immunomodulatory metabolite itaconate modifies NLRP3 and inhibits inflammasome activation. Cell Metab. 2020;32(3):468–478 e7. 32791101 10.1016/j.cmet.2020.07.016 PMC7422798

[184] Mills EL , Ryan DG , Prag HA , Dikovskaya D , Menon D , Zaslona Z , et al. Itaconate is an anti‐inflammatory metabolite that activates Nrf2 via alkylation of KEAP1. Nature. 2018;556(7699):113–117. 29590092 10.1038/nature25986 PMC6047741

[185] Muri J , Wolleb H , Broz P , Carreira EM , Kopf M . Electrophilic Nrf2 activators and itaconate inhibit inflammation at low dose and promote IL‐1β production and inflammatory apoptosis at high dose. Redox Biol. 2020;36:101647. 32863237 10.1016/j.redox.2020.101647 PMC7387846

[186] Alonso‐Pineiro JA , Gonzalez‐Rovira A , Sánchez‐Gomar I , Moreno JA , Durán‐Ruiz MC . Nrf2 and heme Oxygenase‐1 involvement in atherosclerosis related oxidative stress. Antioxidants (Basel). 2021;10(9):1463. 34573095 10.3390/antiox10091463 PMC8466960

[187] He R , Liu B , Xiong R , Geng B , Meng H , Lin W , et al. Itaconate inhibits ferroptosis of macrophage via Nrf2 pathways against sepsis‐induced acute lung injury. Cell Death Discov. 2022;8(1):43. 35110526 10.1038/s41420-021-00807-3 PMC8810876

[188] Pan X , Shan H , Bai J , Gao T , Chen B , Shen Z , et al. Four‐octyl itaconate improves osteoarthritis by enhancing autophagy in chondrocytes via PI3K/AKT/mTOR signalling pathway inhibition. Commun Biol. 2022;5(1):641. 35768581 10.1038/s42003-022-03592-6 PMC9242998

[189] Zhang P , Wang X , Peng Q , Jin Y , Shi G , Fan Z , et al. Four‐octyl itaconate protects chondrocytes against H(2)O(2)‐induced oxidative injury and attenuates osteoarthritis progression by activating Nrf2 signaling. Oxid Med Cell Longev. 2022;2022:2206167. 35126808 10.1155/2022/2206167 PMC8813279

[190] Zhang Q , Bai X , Wang R , Zhao H , Wang L , Liu J , et al. 4‐octyl itaconate inhibits lipopolysaccharide (LPS)‐induced osteoarthritis via activating Nrf2 signalling pathway. J Cell Mol Med. 2022;26(5):1515–1529. 35068055 10.1111/jcmm.17185 PMC8899168

[191] Kon E , Engebretsen L , Verdonk P , Nehrer S , Filardo G . Clinical outcomes of knee osteoarthritis treated with an autologous protein solution injection: a 1‐year pilot double‐blinded randomized controlled trial. Am J Sports Med. 2018;46(1):171–180. 29016185 10.1177/0363546517732734

[192] Everts P , Onishi K , Jayaram P , Lana JF , Mautner K . Platelet‐Rich plasma: new performance understandings and therapeutic considerations in 2020. Int J Mol Sci. 2020;21(20):7794. 10.3390/ijms21207794 PMC7589810 33096812

[193] O'Donnell C , Migliore E , Grandi FC , Koltsov J , Lingampalli N , Cisar C , et al. Platelet‐Rich plasma (PRP) from older males with knee osteoarthritis depresses chondrocyte metabolism and upregulates inflammation. J Orthop Res. 2019;37(8):1760–1770. 31042308 10.1002/jor.24322 PMC6824920

[194] Uchiyama R , Toyoda E , Maehara M , Wasai S , Omura H , Watanabe M , et al. Effect of platelet‐Rich plasma on M1/M2 macrophage polarization. Int J Mol Sci. 2021;22(5):2336. 10.3390/ijms22052336 PMC7956636 33652994

[195] Bennell KL , Hunter DJ , Paterson KL . Platelet‐Rich plasma for the Management of hip and Knee Osteoarthritis. Curr Rheumatol Rep. 2017;19(5):24. 28386761 10.1007/s11926-017-0652-x

[196] Zhang P , Li K , Kamali A , Ziadlou R , Ahmad P , Wang X , et al. Small molecules of herbal origin for osteoarthritis treatment: in vitro and in vivo evidence. Arthritis Res Ther. 2022;24(1):105. 35545776 10.1186/s13075-022-02785-y PMC9092710

[197] Liu FC , Hung LF , Wu WL , Chang DM , Huang CY , Lai JH , et al. Chondroprotective effects and mechanisms of resveratrol in advanced glycation end products‐stimulated chondrocytes. Arthritis Res Ther. 2010;12(5):R167. 20825639 10.1186/ar3127 PMC2990994

[198] Ma P , Yue L , Yang H , Fan Y , Bai J , Li S , et al. Chondroprotective and anti‐inflammatory effects of amurensin H by regulating TLR4/Syk/NF‐kappaB signals. J Cell Mol Med. 2020;24(2):1958–1968. 31876072 10.1111/jcmm.14893 PMC6991675

[199] Wang Z , Huang J , Zhou S , Luo F , Xu W , Wang Q , et al. Anemonin attenuates osteoarthritis progression through inhibiting the activation of IL‐1β/NF‐κB pathway. J Cell Mol Med. 2017;21(12):3231–3243. 28643466 10.1111/jcmm.13227 PMC5706500

[200] Zhang Y , Zeng Y . Curcumin reduces inflammation in knee osteoarthritis rats through blocking TLR4 /MyD88/NF‐kappaB signal pathway. Drug Dev Res. 2019;80(3):353–359. 30663793 10.1002/ddr.21509

[201] Yue B , Ren YJ , Zhang JJ , Luo XP , Yu ZL , Ren GY , et al. Anti‐inflammatory effects of Fargesin on chemically induced inflammatory bowel disease in mice. Molecules. 2018;23(6):1380. 10.3390/molecules23061380 PMC6100621 29880739

[202] Xu X , Zhu R , Ying J , Zhao M , Wu X , Cao G , et al. Nephrotoxicity of herbal medicine and its prevention. Front Pharmacol. 2020;11:569551. 33178019 10.3389/fphar.2020.569551 PMC7593559

[203] Yang M , Jiang L , Wang Q , Chen H , Xu G . Traditional Chinese medicine for knee osteoarthritis: An overview of systematic review. PloS One. 2017;12(12):e0189884. 29267324 10.1371/journal.pone.0189884 PMC5739454

[204] Das P , Jana S , Kumar Nandi S . Biomaterial‐based therapeutic approaches to osteoarthritis and cartilage repair through macrophage polarization. Chem Rec. 2022;22(9):e202200077. 35792527 10.1002/tcr.202200077

[205] Brown BN , Ratner BD , Goodman SB , Amar S , Badylak SF . Macrophage polarization: an opportunity for improved outcomes in biomaterials and regenerative medicine. Biomaterials. 2012;33(15):3792–3802. 22386919 10.1016/j.biomaterials.2012.02.034 PMC3727238

[206] Boersema GS , Grotenhuis N , Bayon Y , Lange JF , Bastiaansen‐Jenniskens YM . The effect of biomaterials used for tissue regeneration purposes on polarization of macrophages. Biores Open Access. 2016;5(1):6–14. 26862468 10.1089/biores.2015.0041 PMC4744891

[207] Harrell CR , Markovic BS , Fellabaum C , Arsenijevic A , Volarevic V . Mesenchymal stem cell‐based therapy of osteoarthritis: current knowledge and future perspectives. Biomed Pharmacother. 2019;109:2318–2326. 30551490 10.1016/j.biopha.2018.11.099

[208] Liang X , Ding Y , Zhang Y , Tse HF , Lian Q . Paracrine mechanisms of mesenchymal stem cell‐based therapy: current status and perspectives. Cell Transplant. 2014;23(9):1045–1059. 23676629 10.3727/096368913X667709

[209] Chen W , Huang Y , Han J , Yu L , Li Y , Lu Z , et al. Immunomodulatory effects of mesenchymal stromal cells‐derived exosome. Immunol Res. 2016;64(4):831–840. 27115513 10.1007/s12026-016-8798-6

[210] Cosenza S , Ruiz M , Toupet K , Jorgensen C , Noël D . Mesenchymal stem cells derived exosomes and microparticles protect cartilage and bone from degradation in osteoarthritis. Sci Rep. 2017;7(1):16214. 29176667 10.1038/s41598-017-15376-8 PMC5701135

[211] Fernandes TL , Gomoll AH , Lattermann C , Hernandez AJ , Bueno DF , Amano MT . Macrophage: a potential target on cartilage regeneration. Front Immunol. 2020;11:111. 32117263 10.3389/fimmu.2020.00111 PMC7026000

[212] Munoz J , Akhavan NS , Mullins AP , Arjmandi BH . Macrophage polarization and osteoporosis: a review. Nutrients. 2020;12(10):2999. 33007863 10.3390/nu12102999 PMC7601854

[213] Ruytinx P , Proost P , van Damme J , Struyf S . Chemokine‐induced macrophage polarization in inflammatory conditions. Front Immunol. 2018;9:1930. 30245686 10.3389/fimmu.2018.01930 PMC6137099

[214] Liang C , Wu S , Xia G , Huang J , Wen Z , Zhang W , et al. Engineered M2a macrophages for the treatment of osteoarthritis. Front Immunol. 2022;13:1054938. 36582221 10.3389/fimmu.2022.1054938 PMC9792488

[215] Wang Y , Li N , Zhang X , Horng T . Mitochondrial metabolism regulates macrophage biology. J Biol Chem. 2021;297(1):100904. 34157289 10.1016/j.jbc.2021.100904 PMC8294576

[216] Jung SB , Choi MJ , Ryu D , Yi HS , Lee SE , Chang JY , et al. Reduced oxidative capacity in macrophages results in systemic insulin resistance. Nat Commun. 2018;9(1):1551. 29674655 10.1038/s41467-018-03998-z PMC5908799

[217] Panahi Y , Alishiri GH , Parvin S , Sahebkar A . Mitigation of systemic oxidative stress by curcuminoids in osteoarthritis: results of a randomized controlled trial. J Diet Suppl. 2016;13(2):209–220. 25688638 10.3109/19390211.2015.1008611

[218] Van den Bossche J , Baardman J , Otto NA , van der Velden S , Neele AE , van den Berg SM , et al. Mitochondrial dysfunction prevents repolarization of inflammatory macrophages. Cell Rep. 2016;17(3):684–696. 27732846 10.1016/j.celrep.2016.09.008

[219] Yuan Y , Chen Y , Peng T , Li L , Zhu W , Liu F , et al. Mitochondrial ROS‐induced lysosomal dysfunction impairs autophagic flux and contributes to M1 macrophage polarization in a diabetic condition. Clin Sci (Lond). 2019;133(15):1759–1777. 31383716 10.1042/CS20190672

[220] Mills CD , Kincaid K , Alt JM , Heilman MJ , Hill AM . M‐1/M‐2 macrophages and the Th1/Th2 paradigm. J Immunol. 2000;164(12):6166–6173. 10843666 10.4049/jimmunol.164.12.6166

[221] Wynn TA , Chawla A , Pollard JW . Macrophage biology in development, homeostasis and disease. Nature. 2013;496(7446):445–455. 23619691 10.1038/nature12034 PMC3725458

[222] Rodríguez‐Morales P , Franklin RA . Macrophage phenotypes and functions: resolving inflammation and restoring homeostasis. Trends Immunol. 2023;44(12):986–998. 37940394 10.1016/j.it.2023.10.004 PMC10841626

[223] Chistiakov DA , Bobryshev YV , Nikiforov NG , Elizova NV , Sobenin IA , Orekhov AN . Macrophage phenotypic plasticity in atherosclerosis: the associated features and the peculiarities of the expression of inflammatory genes. Int J Cardiol. 2015;184:436–445. 25755062 10.1016/j.ijcard.2015.03.055

[224] Liao S , Yang M , Li D , Wu Y , Sun H , Lu J , et al. Comprehensive bulk and single‐cell transcriptome profiling give useful insights into the characteristics of osteoarthritis associated synovial macrophages. Front Immunol. 2022;13:1078414. 36685529 10.3389/fimmu.2022.1078414 PMC9849898

[225] Liu Y , Lu T , Liu Z , Ning W , Li S , Chen Y , et al. Six macrophage‐associated genes in synovium constitute a novel diagnostic signature for osteoarthritis. Front Immunol. 2022;13:936606. 35967352 10.3389/fimmu.2022.936606 PMC9368762

[226] Knights AJ , Farrell EC , Ellis OM , Song MJ , Appleton CT , Maerz T . Synovial macrophage diversity and activation of M‐CSF signaling in post‐traumatic osteoarthritis. bioRxiv; 2023. 10.7554/eLife.93283 PMC11839164 39969512

